# Multi-trait and cross-population genome-wide association studies across autoimmune and allergic diseases identify shared and distinct genetic component

**DOI:** 10.1136/annrheumdis-2022-222460

**Published:** 2022-06-26

**Authors:** Yuya Shirai, Yoshimitsu Nakanishi, Akari Suzuki, Hachirou Konaka, Rika Nishikawa, Kyuto Sonehara, Shinichi Namba, Hiroaki Tanaka, Tatsuo Masuda, Moto Yaga, Shingo Satoh, Mayuko Izumi, Yumiko Mizuno, Tatsunori Jo, Yuichi Maeda, Takuro Nii, Eri Oguro-Igashira, The Biobank Japan Project, Takayuki Morisaki, Yoichiro Kamatani, Shingo Nakayamada, Chikako Nishigori, Yoshiya Tanaka, Yoshito Takeda, Kazuhiko Yamamoto, Atsushi Kumanogoh, Yukinori Okada

**Affiliations:** 1 Department of Statistical Genetics, Osaka University Graduate School of Medicine, Suita, Japan; 2 Department of Respiratory Medicine and Clinical Immunology, Osaka University Graduate School of Medicine, Suita, Japan; 3 Department of Immunopathology, Immunology Frontier Research Center (WPI-IFReC), Osaka University, Suita, Japan, Japan; 4 Integrated Frontier Research for Medical Science Division, Institute for Open and Transdisciplinary Research Initiatives (OTRI), Osaka University, Suita, Japan; 5 Department of Advanced Clinical and Translational Immunology, Osaka University Graduate School of Medicine, Suita, Japan; 6 Laboratory for Autoimmune Diseases, RIKEN Center for Integrative Medical Sciences, Yokohama, Japan; 7 Department of Respiratory Medicine and Clinical Immunology, Public Interest Incorporated Foundation, Nippon Life Saiseikai, Nippon Life Hospital, Osaka, Japan; 8 Division of Dermatology, Department of Internal Related, Kobe University Graduate School of Medicine, Kobe, Japan; 9 The First Department of Internal Medicine, University of Occupational and Environmental Health, School of Medicine, Kitakyushu, Fukuoka, Japan; 10 Department of Obstetrics and Gynecology, Osaka University Graduate School of Medicine, Suita, Japan; 11 StemRIM Institute of Regeneration-Inducing Medicine, Osaka University, Suita, Japan; 12 Laboratory of Immune Regulation, Department of Microbiology and Immunology, Osaka University Graduate School of Medicine, Suita, Japan; 13 Department of Respiratory Medicine, National Hospital Organization Osaka Toneyama Medical Center, Toyonaka, Japan; 14 Institue of Medical Science, The University of Tokyo, Tokyo, Japan; 15 Division of Molecular Pathology, Institute of Medical Science, The University of Tokyo, Tokyo, Japan; 16 Department of Internal Medicine, Institute of Medical Science, The University of Tokyo Hospital, Tokyo, Japan; 17 Laboratory of Complex Trait Genomics, Department of Computational Biology and Medical Sciences, Graduate School of Frontier Sciences, The University of Tokyo, Tokyo, Japan; 18 Center for Infectious Diseases for Education and Research (CiDER), Osaka University, Suita, Japan; 19 Laboratory for Systems Genetics, RIKEN Center for Integrative Medical Sciences, Yokohama, Japan; 20 Laboratory of Statistical Immunology, Immunology Frontier Research Center (WPI-IFReC), Suita, Japan; 21 Department of Genome Informatics, Graduate School of Medicine, The University of Tokyo, Tokyo, Japan

**Keywords:** Arthritis, Rheumatoid, Autoimmune Diseases, Immune Complex Diseases, Lupus Erythematosus, Systemic

## Abstract

**Objectives:**

Autoimmune and allergic diseases are outcomes of the dysregulation of the immune system. Our study aimed to elucidate differences or shared components in genetic backgrounds between autoimmune and allergic diseases.

**Methods:**

We estimated genetic correlation and performed multi-trait and cross-population genome-wide association study (GWAS) meta-analysis of six immune-related diseases: rheumatoid arthritis, Graves’ disease, type 1 diabetes for autoimmune diseases and asthma, atopic dermatitis and pollinosis for allergic diseases. By integrating large-scale biobank resources (Biobank Japan and UK biobank), our study included 105 721 cases and 433 663 controls. Newly identified variants were evaluated in 21 778 cases and 712 767 controls for two additional autoimmune diseases: psoriasis and systemic lupus erythematosus. We performed enrichment analyses of cell types and biological pathways to highlight shared and distinct perspectives.

**Results:**

Autoimmune and allergic diseases were not only mutually classified based on genetic backgrounds but also they had multiple positive genetic correlations beyond the classifications. Multi-trait GWAS meta-analysis newly identified six allergic disease-associated loci. We identified four loci shared between the six autoimmune and allergic diseases (rs10803431 at *PRDM2*, OR=1.07, p=2.3×10^−8^, rs2053062 at *G3BP1*, OR=0.90, p=2.9×10^−8^, rs2210366 at *HBS1L*, OR=1.07, p=2.5×10^−8^ in Japanese and rs4529910 at *POU2AF1*, OR=0.96, p=1.9×10^−10^ across ancestries). Associations of rs10803431 and rs4529910 were confirmed at the two additional autoimmune diseases. Enrichment analysis demonstrated link to T cells, natural killer cells and various cytokine signals, including innate immune pathways.

**Conclusion:**

Our multi-trait and cross-population study should elucidate complex pathogenesis shared components across autoimmune and allergic diseases.

**WHAT IS ALREADY KNOWN ON THIS TOPIC**

- Autoimmune and allergic diseases are distinct outcome of the dysregulation of the immune system, while their differences, or shared components, in genetic backgrounds are elusive.
- The long-term risks of autoimmune diseases are significantly higher in patients with allergic diseases, but the mechanism is unknown.

**WHAT THIS STUDY ADDS**

- Our study clearly depicted distinct disease classifications between autoimmune and allergic diseases due to different polygenic architecture. On the other hand, our study also showed several multiple positive genetic correlations beyond the classifications.
- Our multi-trait and cross-population analysis identified four loci shared between autoimmune and allergic diseases (*PRDM2*, *HBS1L*, *G3BP1* and *POU2AF1*), which showed population-specific or cross-populational effects. Such shared loci were characterised as associations with genes involved in innate immunity or humoral immunity.

**HOW THIS STUDY MIGHT AFFECT RESEARCH, PRACTICE AND/OR POLICY**

- The shared effects identified in this study may be responsible for both autoimmune and allergic diseases. Our multi-trait approach proposes effective strategies to identifying shared genetic components, which contributes to understanding a set of complex human traits such as immune-related diseases.

## Introduction

Genetic background contributes to the development of common and complex diseases, and genome-wide association studies (GWASs) have identified a number of genetic loci that affect a variety of disease risk.^1^ Genetic backgrounds of diseases can be decomposed into disease-specific effects and those shared across diseases. While understanding disease-specific effects helps us comprehend the individual disease pathologies, understanding shared effects is also important to reveal underlying pathologies across diseases and provide opportunities for reciprocal drug repositioning. Previous GWAS integrating allergic diseases have revealed their shared genetic background among allergic diseases (eg, asthma, pollinosis (PO) and eczema^2 3^). Autoimmune diseases are another outcome of dysregulation of the immune system. Several GWASs dealing with multiple autoimmune diseases successfully identified the genetic overlap existing in autoimmunity.^4–6^ By integrating similar diseases, these studies have advanced the knowledge of the shared aetiology in each immune dysfunction. While autoimmune and allergic diseases are pathogenetically distinct conditions, several elements such as antibodies, T cells, mast cells and cytokines are involved in both.^7^ Furthermore, several allergic diseases are associated with the long-term risks of developing autoimmune diseases.^8^ These observations suggest shared genetic components across autoimmune and allergic diseases, but there have been few genetic studies that conducted multi-trait integrative analysis. Furthermore, majority of such approaches focused on a single ancestry, thereby lacking global landscape of human disease genetics.

Biobanks have been accumulating genotypes and medical records on a huge scale,^9 10^ including autoimmune and allergic diseases, which encourage us to elucidate the genetic background of immune dysfunction. In this study, we estimated the genetic correlations among three autoimmune (rheumatoid arthritis (RA), Graves’ disease (GD) and type 1 diabetes (T1D)) and three allergic diseases (bronchial asthma (BA), PO and atopic dermatitis (AD)) by using the BioBank Japan (BBJ) and UK Biobank (UKB) resources.^11 12^ To identify shared genetic components, we conducted multi-trait and cross-population meta-analyses integrating the GWAS datasets. We further performed enrichment analyses of cell types and biological pathways to highlight shared and distinct perspectives in biological functions.

## Methods

### Study cohorts and subjects

All the Japanese subjects enrolled in this study were collected through BBJ, which is a hospital-based registry with multiomics data from genotype to multitude phenotype of approximately 200 000 patients with 1 of 47 diseases.^11^ We extracted the subjects with autoimmune and allergic diseases registered in BBJ, which composed of AD (2472 cases), BA (7522 cases), GD (2041 cases), PO (5308 cases), anticyclic citrullinated peptide-positive RA (2370 cases) and T1D without a record of type 2 diabetes (638 cases). The controls were the subjects without medical records of any immune-related diseases.

For the European subjects, we obtained the data of UKB, which is a population-based registry on approximately 500 000 individuals aged between 40 and 69 recruited in the UK.^12^ Analogous to BBJ, we selected the six autoimmune and allergic diseases as the following definition. AD cases were the subjects registered as AD in hospital records or eczema/dermatitis in self-reported diagnosis (12 285 cases). BA cases were the subjects registered as asthma in either hospital records or self-reported diagnosis (54 872 cases). GD cases were the subjects registered as thyrotoxicosis with diffuse goitre in hospital records or GD in self-reported diagnosis (614 cases). PO cases were the subjects registered as allergic rhinitis due to pollen in hospital records or hayfever/allergic rhinitis in self-reported diagnosis (26 758 cases). RA cases were the subjects registered as seropositive RA in hospital records or RA in self-reported diagnosis (5065 cases). T1D cases were registered as insulin-dependent diabetes mellitus in hospital records or T1D in self-reported diagnosis without the following medical records: insulin-independent diabetes mellitus in hospital records, type 2 diabetes mellitus or gestational diabetes mellitus in self-reported diagnosis (914 cases). The controls were subjects with no records of any immune-related diseases in hospital records or self-reported diagnosis.

The summary of the study cohorts and subjects is described in online supplemental table 1. All the subjects agreed with informed consent based on the approval of the institutional ethical committee. This study was approved by the ethical committee of Osaka University (Approval ID: 734–14).

10.1136/annrheumdis-2022-222460.supp1 Supplementary data

### Genotyping and imputation

The BBJ subjects were genotyped with the Illumina HumanOmniExpressExome BeadChip or a combination of the Illumina HumanOmniExpress and HumanExome BeadChips.^13^ Quality control of participants and genotypes was performed as described elsewhere.^14^ In this study, we extracted East Asian subjects based on a principal components analysis of the genotypes. We performed haplotype phasing of the genotype data using Eagle (V.2.3) and imputed genotype dosages using Minimac V.3 with the population-specific reference panel of Japanese, which was integrated whole-genome sequence data of 1000 Genomes Project Phase 3 (V.5) and 1037 Japanese.^15^

The UKB subjects were genotyped with the Applied Biosystems UK BiLEVE Axiom Array or the Applied Biosystems UKB Axiom Array. After quality control as described elsewhere,^10^ haplotype phasing was performed using SHAPEIT3 and genotype dosages were imputed using IMPUTE4 with the merged UK10K and 1000 Genomes phase 3 reference panels. We extracted Caucasian subjects based on a principal components analysis of the genotypes for subsequent analysis.

### Individual-trait GWAS

We performed a GWAS for the individual autoimmune and allergic disease with a generalised linear mixed model implemented in SAIGE.^16^ Age, sex and the top five principal components were included as covariates in the regression model. We applied the leave-one-chromosome-out approach to calculate the genetic relation matrix. We excluded the variants with either imputation quality *Rsq* <0.7, minor allele frequency <0.005 or minor allele count<3 from the GWAS. The genome-wide significance threshold was adopted at the level of p=5.0 × 10^−8^. We considered the human leucocyte antigen (HLA) region (chr6:26Mb-34Mb) as one locus considering its complex and strong linkage disequilibrium (LD) structure within the region.^13^

### Heritability and genetic correlation

We estimated heritability and confounding bias for the individual traits using LD score regression (LDSC) analysis^17^ with 1000 Genomes phase 3 East Asian (1000G-EAS) reference panel for the BBJ GWAS data sets and 1000 Genomes phase 3 European (1000G-EUR) reference panel for the UKB GWAS data sets. To assess genetic correlations among the six autoimmune or allergic diseases, we used high-definition likelihood (HDL) inference,^18^ which is an extension of LDSC in that it thoroughly exploits the information of the variance–covariance matrix of the *Z*-score from GWAS summary statistics. Because HDL needed a larger reference sample for accurate estimation than LDSC, we prepared a custom reference panel from 1000G-EAS and BBJ genotype data to analyse the BBJ GWAS data sets. We used the prebuilding UKB reference panel to analyse the UKB GWAS data sets. We excluded the variants within the HLA region for the estimation in both LDSC and HDL. Hierarchical clustering for the genetic correlation matrix was performed with Ward’s method using 1 - *r_g_* as distance metrics.

### Local heritability and genetic correlation

We applied SUPERGNOVA^19^ to estimate local heritability and genetic covariance in the prespecified LD-independent segments by ldetect.^20^ While SUPERGNOVA can effectively estimate local genetic covariance accounting for sample overlap, local genetic correlation estimates are numerically unstable due to the noise in the estimates of local heritability. We assessed the significance of the local genetic correlations based on the significance of local genetic covariances as was done in the paper of SUPERGNOVA because they are statistically equivalent.

### Meta-analysis for autoimmune and allergic diseases

We conducted fixed effect meta-analyses with the Lin-Sullivan method,^21^ taking into account sample overlap among GWAS data sets. To account for the effects of heterogeneity, we applied Metasoft to calculate heterogeneity index *I^2^* and p value based on Cochran’s Q test (*P_het_*). When heterogeneity was suggested (*I^2^* ≧ 50 or *P_het_* <0.05), we prioritised the p value in the random effect model calculated with RE2C.^22^ First, we performed two types of meta-analyses that integrated three autoimmune diseases or three allergic diseases GWAS data sets. Second, we performed a multi-trait meta-analysis integrating six GWAS data sets. Finally, we performed a cross-population meta-analysis that integrated all of the 12 GWAS data sets. We calculated the genomic control factor *λ_GC_* using R statistical software. Genome-wide significance threshold was adopted at the level of p=5.0×10^−8^. We applied FUMA^23^ to define independent associated loci using the default *r^2^* threshold. As the LD reference panel for FUMA, we referred to 1000G-EAS reference panel for the BBJ meta-analysis and 1000G-EUR reference panel for the UKB meta-analysis. For the cross-population meta-analysis, we referred 1000G-ALL reference panel, which is the only available cross-population LD reference panel in FUMA. We defined a novel locus if all the variants and genes in identified loci were not associated with diseases included in the meta-analysis by querying GWAS catalogue,^24^ PheWeb,^25^ PheWeb.jp,^3^ PhenoScanner (v2)^26^ and Open Targets Genetics.^27^ We additionally defined an independent locus if a lead variant was located in previously reported genes but not LD (*r* ^2^ <0.1) with the reported variants. We created regional plots using LocusZoom for novel and independent loci.

### Fine-mapping and functional annotation

We used SuSiE^28^ to find 95% credible sets of causal variants accounting for LD in the loci identified in our study. In SuSiE, the LD information was referred to the 1000G-EAS and BBJ reference panel for the BBJ meta-analysis, the 1000G-EUR reference panel for the UKB meta-analysis and the reference panel integrating 1000G-EAS and 1000G-EUR for the cross-population meta-analysis. We obtained functional annotations of the lead variants using ANNOVAR.^29^ Annotation of promotor and enhancer marks for the individual lead variants were searched through HaploReg (V.4.1). Quantitative effects on gene expression levels of the variants (ie, eQTL effect) were queried according to GTEx Portal (V.8)^30^ and ImmuNexUT,^31^ that is the latest eQTL data set of 28 immune cells in Japanese population. Because we could access the summary statistics of ImmuNexUT, we performed colocalisation analysis using eCAVIAR^32^ to assess the sharing causal variants between the BBJ GWAS data sets and ImmuNexUT eQTL data sets. We set CLPP ≧0.03 as a threshold for significant colocalisation as was done in the paper of ImmuNexUT.

### Cell-type enrichment analysis

To assess the enrichment of the autoimmune and allergic GWAS data sets in immune cell types, we used stratified LDSC^33^ for the gene annotations with the highest specific expression in 292 immune cell types from the ImmGen Consortium.^34^ We used the 1000G–EAS and 1000G–EUR baseline V.1.2 LD score in BBJ and UKB, respectively, and excluded the variants within the HLA region from the analysis. We calculated the p value of the regression coefficient τ_c_ of the individual annotation. We set the threshold for significant enrichment as p=0.05/292, adjusted by Bonferroni correction. We performed hierarchical clustering on the matrix of enrichment significance in the 292 cell-type-specific annotations, using Euclidean distance and Ward’s method.

### Pathway enrichment analysis

We evaluated the association between the GWAS data sets and molecular pathways using PASCAL.^35^ PASCAL calculates gene-based scores by integrating p values of variants and estimate pathway enrichment scores by merging gene-based scores belonging to the same pathway. As the reference panel, we used the custom 1000G-EAS genotype data for the BBJ GWAS data sets and the 1000G-EUR genotype data provided by the authors for the UKB GWAS data sets. To assess the enrichment within the immune pathway, we obtained the curated gene sets derived from the Reactome pathway database in MSigDB collections^36^ and extracted 150 gene sets in the lower layers of ‘immune system’. We set the threshold for significant enrichment as p=0.05/150, adjusted by Bonferroni correction. For the visualisation of the enriched pathways, we used Cytoscape^37^ to create a network diagram.

### Replication analysis for additional autoimmune diseases

We additionally evaluated the association of the four variants newly identified in the multi-trait analysis of the six autoimmune and allergic diseases with two additional autoimmune diseases: psoriasis (PsO) and systemic lupus erythematosus (SLE). We meta-analysed overall 11 807 cases and 696 291 controls in PsO and 9987 cases and 712 510 controls in SLE. For the EAS cohort, we used the imputed dosage data of the subjects in BBJ, Osaka University Graduate School of Medicine and previous GWAS summary statistics.^38^ For the EUR cohort, we used the imputed dosage data of UKB and previous GWAS summary statistics.^39^ The summary of the data sets for the replication analysis is described in online supplemental table 2.

As for the dosage data, we performed association analyses for the individual data set using SAIGE in the same condition as our GWAS. Subsequently, we integrated the summary statistics with Metasoft for each disease in the population-specific and cross-population manner. Finally, we conducted multi-trait meta-analyses with RE2C, dealing with sample overlap.

### Drug target analysis

We queried the genes associated with autoimmune and allergic diseases to STRING V.11.5,^40^ a database that collected protein–protein interaction (PPI) networks. In STRING, each PPI is annotated with a score between 0 and 1 based on physical and functional information. Biologically related neighbourhood genes were defined as genes with a high confidence score (combined score excluding ‘text mining score’ >0.7) to the queried target genes. We confirmed whether the target and neighbourhood genes were drug targets by searching in DrugBank^41^ and Therapeutic Target Database (TTD).^42^

### Patient and public involvement

This research was done without patient and public involvement. Patients and public were not invited to comment on the study design and were not consulted to develop patient relevant outcomes or interpret the results.

## Results

### Overview of the subjects

Our study focused on six immune-related diseases included in BBJ target diseases. In UKB, we extracted the six autoimmune and allergic diseases corresponding to the BBJ target diseases. The autoimmune diseases consisted of RA (2370 cases in BBJ and 5065 cases in UKB), GD (2041 cases in BBJ and 614 cases in UKB) and T1D (638 cases in BBJ and 914 cases in UKB). The allergic diseases consisted of BA (7522 cases in BBJ and 54 872 cases in UKB), AD (2472 cases in BBJ and 12 285 cases in UKB) and PO (5308 cases in BBJ and 26 758 cases in UKB). We enrolled subjects with no records of any immune-related diseases as control (142 192 controls in BBJ and 291 471 controls in UKB). To enhance power to detect the associated loci, we excluded immune-related diseases from the controls. The summary of the subjects is described in online supplemental table 1.

### Individual-trait GWAS analysis in a single ancestry

First, we separately performed a GWAS of individual disease in each ancestry to overview their genetic architecture prior to the meta-analysis. Through the GWASs in BBJ, we observed 4 significant loci in RA, 9 in GD, 1 in T1D, 8 in BA and 9 in AD (online supplemental table 1). Through the GWASs in UKB, we observed 6 significant loci in RA, 2 in GD, 3 in T1D, 88 in BA, 17 in AD and 34 in PO. Although we found no novel loci in the individual-trait GWAS in a single ancestry, all the significant loci were robustly concordant with the previous findings.^3 24–27^

### Global genetic relationships across immune-related diseases

**Figure 1:**
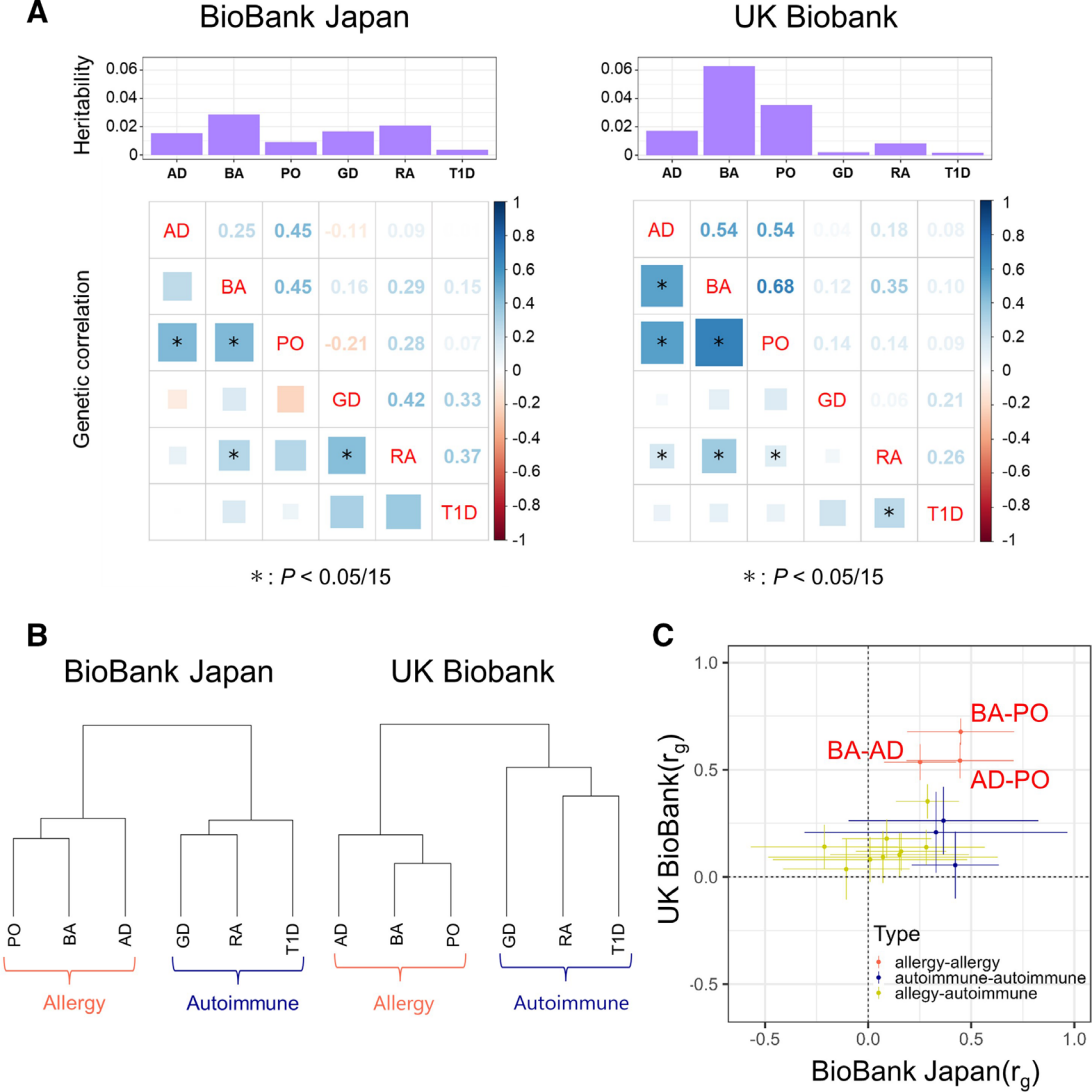
Genetic correlation among the autoimmune and allergic diseases. (A) Histograms of the heritability and genetic correlation matrices of autoimmune and allergic diseases in BBJ (left panel) and UKB (right panel). (B) Dendrograms of the hierarchical clustering based on the genetic correlations in BBJ (left panel) and UKB (right panel). (C) Scatter plots describing the associations between the genetic correlations in BBJ (x-axis) and UKB (y-axis). Dots represent the estimates of the genetic correlation and whiskers represent 95% confidence intervals. The dots are coloured according to disease categories. AD, atopic dermatitis; BA, bronchial asthma; BBJ, BioBank Japan; GD, Grave’s diseases; PO, pollinosis; RA, rheumatoid arthritis; T1D, type 1 diabetes; UKB, UK Biobank.

We applied LDSC to estimate the heritability of the individual GWAS data sets.^17^ The heritability was relatively larger in allergic diseases than in autoimmune diseases (on average, 1.8% in BBJ and 3.8% in UKB for allergic diseases, but 1.4% in BBJ and 0.4% in UKB for autoimmune diseases; figure 1A), although the relatively limited sample sizes and the exclusion of the HLA region in the LDSC framework may have affected the results. Estimates of heritability in the absence of the HLA regions can be underestimated, especially in autoimmune diseases. To finely conduct the subsequent meta-analysis, we applied HDL to more accurately estimate the genetic correlations to find the disease pairs with similar genetic backgrounds.^18^ Our genetic correlation analysis showed that the six immune-related diseases could be divided into the two major categories, which corresponded to the original classifications of autoimmune and allergic diseases. Hierarchical clustering based on genetic correlation clearly described these two major categories (figure 1B). Thus, the genetics-based classification of diseases was consistent with the clinical classification. Larger genetic correlation (*r_g_*) estimates were observed among allergic diseases, suggesting close relationship of genetic backgrounds of the allergic diseases assessed in this study. On the other hand, several disease pairs showed a positive genetic correlation across categories, such as RA and BA in BBJ (*r_g_* =0.29, p=2.2×10^−4^) and UKB (*r_g_* =0.35, p=3.6×10^−18^). We note that the *r_g_* estimates were generally concordant between BBJ and UKB (*r*=0.58, p=0.022; figure 1C), indicating the robustness of our assessments.

### Local genetic relationships across immune-related diseases

To identify local genetic architecture underlying between two disease categories, we applied SUPERGNOVA to estimate the local heritability and the local genetic correlation per LD-independent segment.^19^ In the autoimmune diseases, the local heritability was prominent in the HLA region (online supplemental figures 1 and 2), where strong genetic risk was embedded.^13^ In contrast, the local heritability was distributed relatively across genome-wide in the allergic diseases.

In the local genetic correlation analysis, we found multiple regions with positive correlations within allergic diseases in the UKB data sets (online supplemental figure 3). Notably, there were 38 positively correlated regions between BA and PO, suggesting their shared genetic structure in a genome-wide manner. We also observed several genetic regions with positive genetic correlations across the disease categories. Of these, *CLEC16A* at 16p13 was the hub region where nine loci pairs with positive correlations were centralised (online supplemental figure 4). We obtained less evidence for the local genetic correlation in BBJ than UKB, probably reflecting the difference of the sample sizes in the original GWASs.

### Multi-trait and cross-population meta-analysis within autoimmune or allergic disease categories

**Figure 2:**
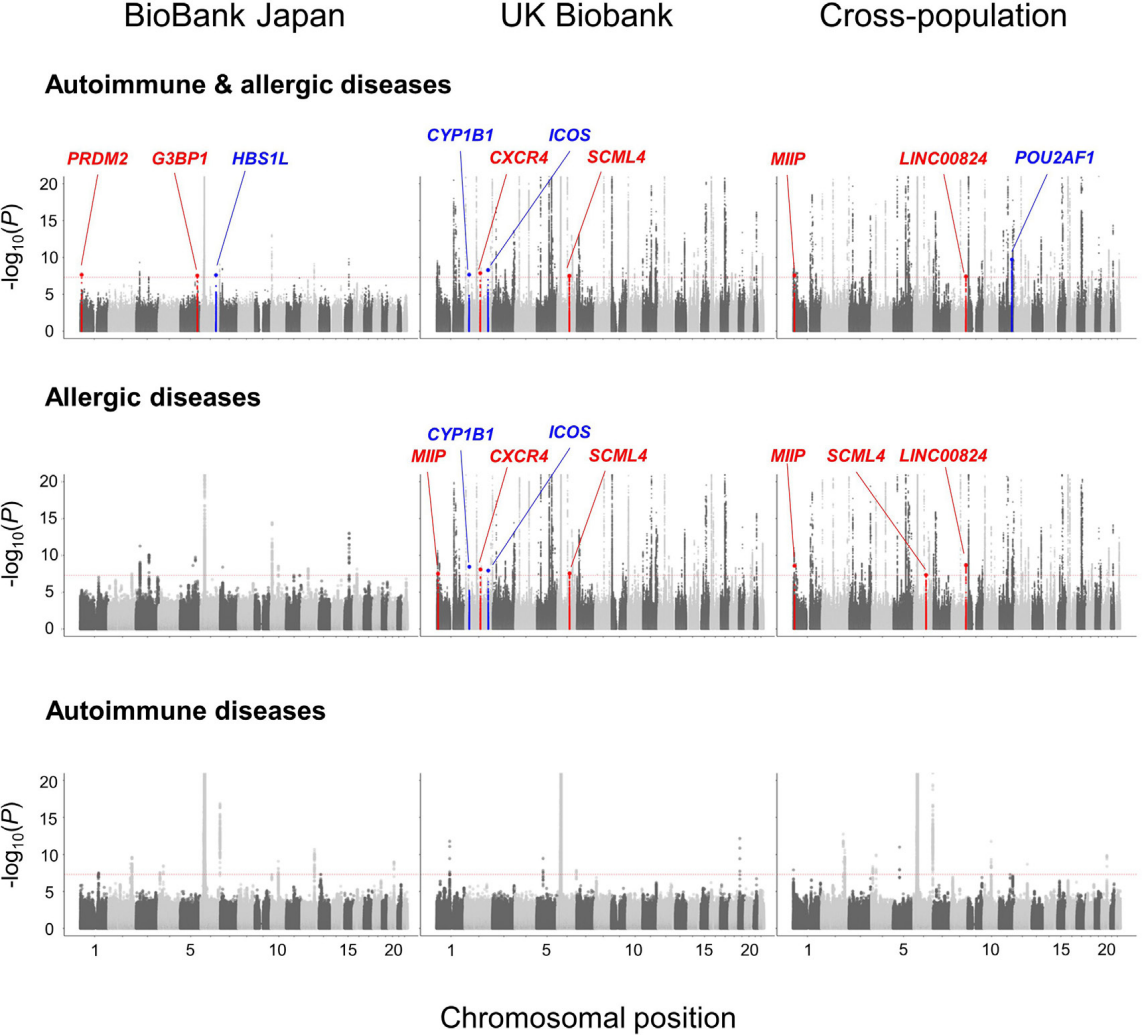
Manhattan plots of the GWAS meta-analysis for the autoimmune and allergic diseases. Manhattan plots of the GWAS meta-analysis of six autoimmune and allergic diseases (upper), three allergic diseases (middle) and three autoimmune diseases (lower). The y-axis indicates −log_10_(*P*) of association of each variant calculated in three cohorts: BBJ (left), UKB (middle), cross-population (right). The upper limit is set to 20 for the sake of clarity. In the loci which we identified, the novel ones are coloured in red and independent ones are coloured in blue. The horizontal dashed red line indicates the genome-wide significance threshold (p=5.0 × 10^−8^). BBJ, BioBank Japan; GWAS, genome-wide association study; UKB, UK Biobank.

We then performed multi-trait GWAS meta-analyses to evaluate the shared effect among GWAS data sets at the variant level, while local genetic analysis helped us assess prespecified independent regions. Because we expected that statistical power would be enhanced by considering diseases with a shared genetic background together, we first conducted a meta-analysis within each disease category separately (figure 2). In the meta-analysis of autoimmune diseases, we tested 8 371 232 variants in the BBJ data sets, 10 862057 variants in the UKB data sets and 5 965 647 variants in the cross-population data sets. In the meta-analysis of the allergic diseases, we tested 8 368 683 variants in the BBJ data sets, 10 856683 variants in the UKB data sets and 5 965 021 variants in the cross-population data sets. While we observed slight inflation of the genomic control factor (*λ_GC_*) in each meta-analysis, LDSC intercept did not obviously deviate from 1.00, suggesting no apparent bias due to confounding population structure (online supplemental figure 5).

In the meta-analysis of the autoimmune diseases, we identified 10, 5 and 11 significant loci in the BBJ, UKB and cross-population data sets, respectively. In the meta-analysis of the allergic diseases, we identified 11, 98 and 99 significant loci in the BBJ, UKB and cross-population data sets, respectively. We found no novel significant loci in the meta-analyses of the autoimmune diseases. On the other hand, we identified three novel loci (rs74052928 G>C at 1p36, *MIIP*, p=3.0×10^−8^; rs575879774 G>GA at 2q21, *CXCR4*, p=8.4×10^−9^ and rs7773622 C>T at 6q21, *SCML4*, p=2.8×10^−8^) and two independent novel association signals within the previously reported loci (rs1800440 T>C at 2p22, *CYP1B1*, p=3.6×10^−9^; rs115257668 A>G at 2q33, *ICOS*, p=1.2×10^−8^) in the UKB meta-analysis of the allergic diseases (table 1).

**Table 1:** Summary of the multi-trait meta-analyses

Locus discovery	Cohort	Chr:position	SNP	Gene	Annotation	BioBank Japan					UK BioBank					Cross-population				
						EAF (control)	OR	P*	I^2^	P_het_†	EAF (control)	OR	P*	I*^2^*	P_het_†	EAF (control)	OR	P*	I^2^	P_het_†
Meta-analysis of allergic diseases																				
Novel	UKB	1:12 080 122	rs74052928	*MIIP*	Intron	0.094	0.95	0.029	31	0.24	0.14	0.95	3.0×10^−8^	0	0.76	0.13	0.95	2.6×10^−9^	0	0.63
Novel	UKB	2:136 809 603	rs575879774	*CXCR4*	Intergenic	0.28	1.01	0.46	0	0.78	0.015	1.16	8.4×10^−9^	0	0.49	0.10	1.05	3.4×10^−4^	83	1.8×10^−5^
Novel	UKB	6:108 131 958	rs7773622	*SCML4*	Intron	0.086	1.01	0.81	67	0.047	0.16	0.96	2.8×10^−8^	0	0.92	0.13	0.96	2.5×10^−7^	54	0.053
Novel	Cross-population	8:129 428 433	rs16902902	*LINC00824*	Intron	0.33	0.93	6.8×10^−7^	66	0.051	0.037	0.95	6.2×10^−4^	0	0.89	0.13	0.94	2.1×10^−9^	27	0.23
Known ‡	UKB	2:38 298 139	rs1800440	*CYP1B1*	Missense	–	–	–	–	–	0.18	1.05	3.6×10^−9^	0	0.38	–	–	–	–	–
Known ‡	UKB	2:205 032 379	rs115257668	*ICOS*	Intergenic	–	–	–	–	–	0.018	1.14	1.2×10^−8^	0	0.84	–	–	–	–	–
Meta-analysis of autoimmune and allergic diseases																				
Novel	BBJ	1:14 206 917	rs10803431	*PRDM2*	Intergenic	0.49	1.07	2.3×10^−8^	3	0.39	0.37	1.00	0.42	0	0.96	0.41	1.01	0.058	73	2.7×10^−5^
Novel	BBJ	5:151 169 881	rs2053062	*G3BP1*	Intron	0.11	0.90	2.9×10^−8^	0	0.54	–	–	–	–	–	–	–	–	–	–
Known ‡	BBJ	6:135 415 208	rs2210366	*HBS1L*	Intron	0.36	1.07	2.5×10^−8^	40	0.14	0.25	1.02	0.03	0	0.62	0.29	1.03	2.9×10^−6^	62	0.0021
Known ‡	Cross-population	11:111 243 102	rs4529910	*POU2AF1*	Intron	0.41	0.96	8.3×10^−4^	71	0.0044	0.73	0.96	5.7×10^−8^	25	0.25	0.63	0.96	1.9×10^−10^	54	0.014

*P-value in the fixed effect model by Lin-Sullivan method.

†P-value based on Cochran’s Q test.

‡Newly identified risk variant independent of the previously known risk variant within the locus.

BBJ, BioBank Japan; Chr, chromosome; EAF, effect allele frequency; SNP, single nucleotide polymorphism; UKB, UK Biobank.

**Figure 3:**
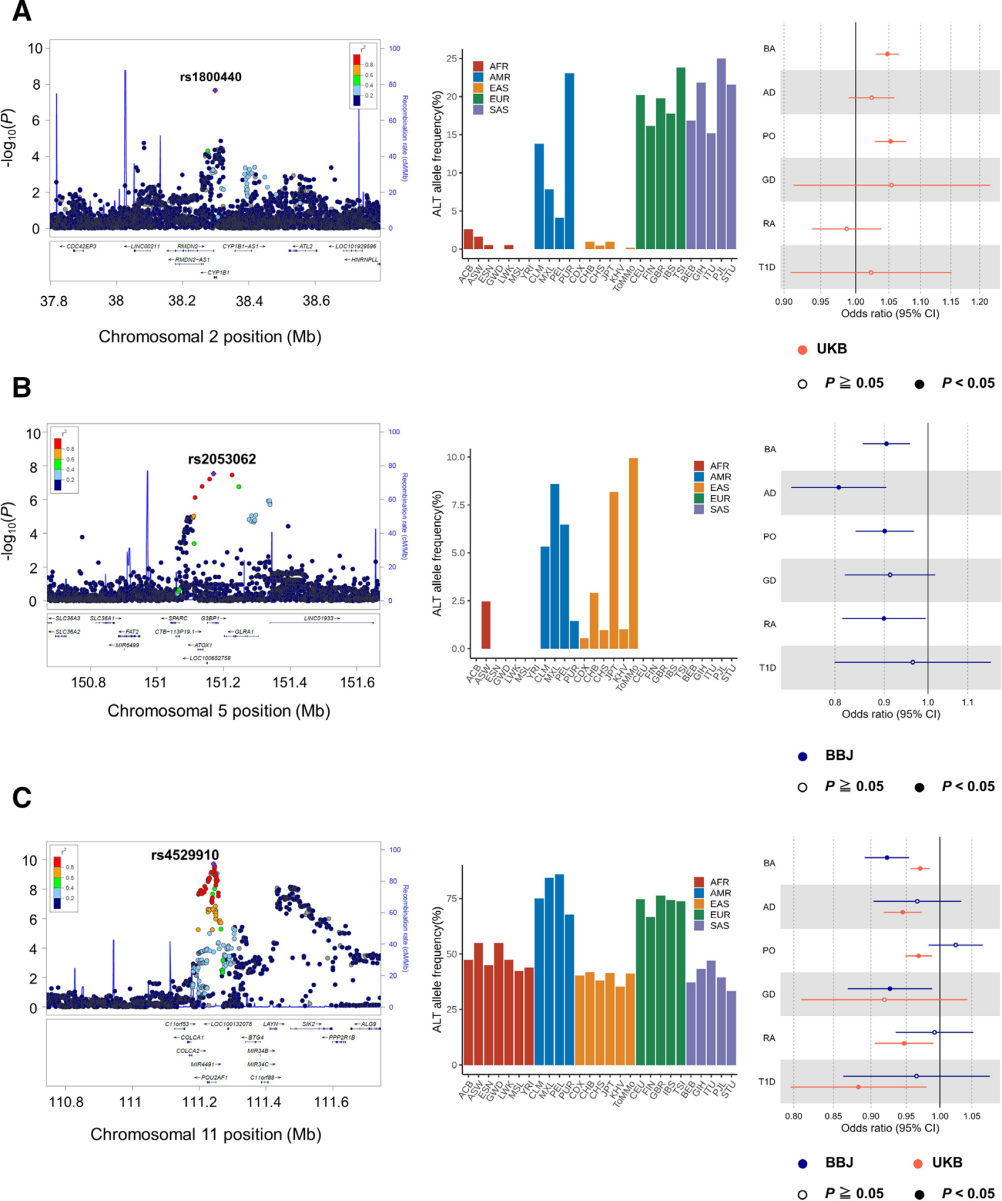
Population-specific and cross-populational disease-associated loci. (A) *CYP1B1* locus, observed in only the UKB datasets, (B) *G3BP1* locus, observed in only the BBJ datasets, and (C) *POU2AF1* locus, observed consistent effect in both ancestries are described as follows. (Left) Regional plot of the individual locus. The lead variants are coloured in purple and all the other variants are coloured based on LD with the lead variant as in the legend. (Middle) Histograms of the alternative allele frequency of the lead variants, which are coloured according to continental populations. (right) Forest plot of the individual lead variants. The dots indicate the OR in each dataset and the whiskers represent 95% confidence intervals. AD, atopic dermatitis; BA, bronchial asthma; BBJ, BioBank Japan; GD, Grave’s diseases; PO, pollinosis; RA, rheumatoid arthritis; T1D, type 1 diabetes; UKB, UK Biobank.

Among the five lead variants, rs1800440 was a missense variant of *CYP1B1*, where the alternative allele was only observed in the UKB data sets (figure 3A). In the statistical fine mapping of putatively causal variants by SuSiE,^28^ the 95% credible set included only rs1800440, which supported that rs1800440 was causal in the loci (online supplemental figure 6). The directional effects of the risk allele of rs1800440-C were concordant among the three allergic diseases, demonstrating nominal association significance in BA and PO (p<0.05). Pathogenicity scores supported that this missense mutation was constrained (GERP ++score = 5.95) and deleterious to human health (CADD=21.8).

We found an additional novel locus in the cross-population meta-analysis of allergic diseases (rs16902902 G>A at 8q24, *LINC00824*, p=2.1×10^−9^). The allele of rs16902902-A was suggested to have a protective effect for allergic diseases in the BBJ data set (p=6.8×10^−7^) and the UKB data set (p=6.2×10^−4^) and exceeded the genome-wide significance level in the cross-population meta-analysis. None of the identified variants showed apparent heterogeneity (*I^2^* <30% and *P_het_* >0.2).

### Multi-trait meta-analysis of the autoimmune and allergic diseases

Our genetic correlation analysis showed cross-category correlations like RA and BA. This suggested that common genetic elements cause both autoimmune and allergic diseases. Thus, we conducted a cross-trait meta-analysis integrating the six GWAS datasets of autoimmune and allergic diseases, first in a single ancestry manner.

In the BBJ GWAS meta-analysis, we identified 10 significant loci, including two novel loci (rs10803431 G>C at 1p36, *PRDM2*, p=2.3×10^−8^; rs2053062 C>T at 5q33, *G3BP1*, p=2.9×10^−8^) and one independent locus (rs2210366 G>A at 6q23, *HBS1L*, p=2.5×10^−8^). Although the variants were nominally but not genome-wide significant in the individual analysis, they became significant after integrating the six GWAS data sets (online supplemental figure 7). The minor allele frequencies of the three lead single-nucleotide polymorphisms (SNPs) were higher in non-Europeans. Especially, rs2053062-T was specific to East Asians (mainly Japanese) and Americans but not included in the UKB data set (figure 3B), highlighting population-specific disease genetic architecture.

The lead SNP of rs2053062 was the *G3BP1* intron variant. The directional effects of the protective allele of rs2053062-T were concordant among the six immune-related diseases, demonstrating nominal association significance in BA, AD, PO and RA. We evaluated the positional overlap between rs2053062 and cell type-specific chromatin states with Haploreg. The variant was located in a region considered to be an enhancer, which was supported by multiple Chip-seq data for T cells. Furthermore, the protective allele of rs2053062-T has been reported as an eQTL that decreases *G3BP1* expression levels in effector regulatory T cells in ImmunNexUT database^31^ (online supplemental figure 8). Our colocalisation analysis supported that rs2053062 affected both the disease risk and the expression levels of *G3BP1* in various lymphocyte cell types (online supplemental figure 9), proposing the expression level as an endophenotype to disease susceptibility.

We identified 98 significant loci in the UKB GWAS meta-analysis, but no novel loci were identified in addition to the meta-analysis of allergic diseases.

Finally, we performed a cross-population meta-analysis integrating the 12 GWAS data sets obtained from the BBJ and UKB. We identified 90 lead variants, one of which was an independent variant that newly satisfied genome-wide significance level (rs4529910 T>G at 11q23, *POU2AF1*, p=1.9×10^−10^). Because the effect of rs4529910 was suggested to be heterogeneous (*I^2^* =53.5% and *P_het_* =0.014), we re-evaluated the association of rs4529910 in the random effect model. Consequently, we observed a more robust association of rs4529910 with the autoimmune and allergic diseases (p=5.8×10^−11^). The lead SNP of rs4529910 was the *POU2AF1* intron variant. Several variants around *POU2AF1* had been reported to be associated with the allergic diseases, including BA, PO and AD. However, these known variants were not in LD (*r^2^* <0.1) with the newly identified risk variant of rs4529910. The statistical fine-mapping analysis by SuSiE described that there were two distinct signals in the loci, which indicated that rs4529910 had a different genetic effect from the reported ones (online supplemental figure 6). The effect allele of rs4529910-G was protectively associated with autoimmune and allergic diseases across ancestries, except for the BBJ PO data set. In Haploreg, the variant was located in a region considered to be an enhancer, which was supported by several Chip-seq data for B cells. The protective allele of rs4529910-G has been reported as an eQTL that decreases *POU2AF1* expression levels in B cells in the ImmunNexUT database (online supplemental figure 10).

### Cell-type enrichment in the autoimmune and allergic diseases

**Figure 4:**
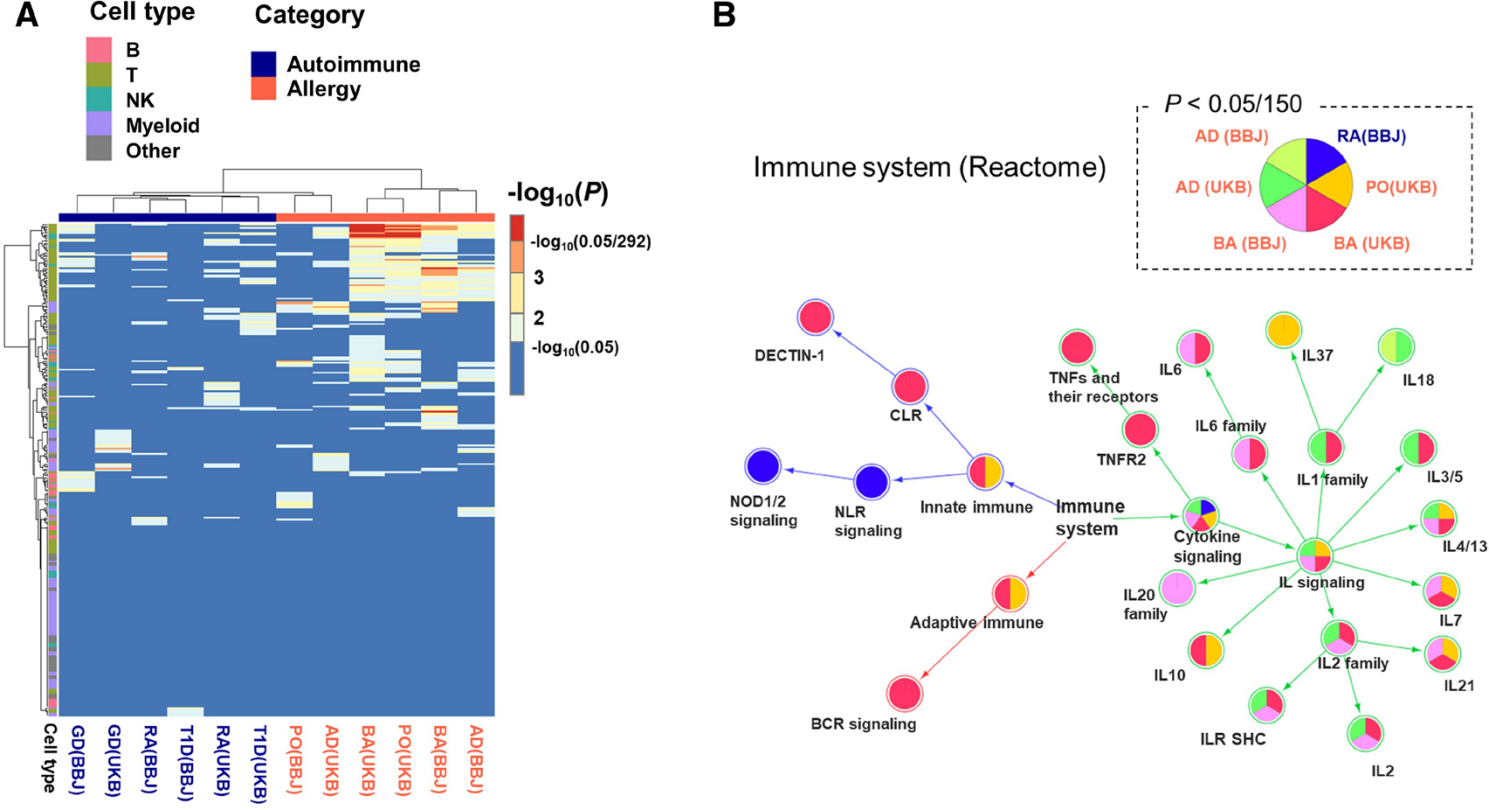
Enrichment analysis for six autoimmune and allergic diseases. (A) A heatmap describing the 292 immune cell-type enrichment using LDSC referring ImmGen gene expression data. The row and column are hierarchically clustered. The row annotations are coloured based on five cell types (B cell, T cell, NK cell, myeloid cell, and others), and the column annotations are coloured according to whether it is an autoimmune or an allergic disease. (B) The pathway network of immune system in Reactome database. The nodes are coloured according to whether the individual GWAS data are significantly enriched at a significance level of 0.05/150. For the sake of clarity, only nodes that have at least one enriched disease are shown. AD, atopic dermatitis; BA, bronchial asthma; BBJ, BioBank Japan; GD, Grave’s diseases; GWAS, genome-wide association study; LDSC, linkage disequilibrium score regression; PO, pollinosis; RA, rheumatoid arthritis; T1D, type 1 diabetes; UKB, UK Biobank.

Our local heritability analysis suggested that the two disease categories were characterised by the different distribution of genetic risk on the genome. To interpret the biological consequences, we performed the enrichment analysis with the 292 immune cell types in ImmGen data set.^34^ Many T cell and natural killer cell subsets were associated with BA or PO at the nominal significance level (figure 4A). Among them, regulatory T and natural killer T cells were significantly enriched in both BA and PO in UKB even after multiple testing correction (table 2). We observed no significant enrichment in the autoimmune diseases potentially due to biased polygenicity resulting from the centralisation of heritability on the HLA region.

**Table 2:** Significantly enriched cell type in autoimmune and allergic diseases

Category	Cell type	P value		
		BA (UKB)	PO (UKB)	BA (BBJ)
T cell	T.4Mem.Sp	**3.8×10^-6^**	2.5×10^-4^	0.0015
T cell	T.4Mem44h62l.Sp	**1.3×10^-5^**	1.9×10^-4^	5.3×10^-4^
Natural killer cell	NKT.4-.Sp	**3.1×10^-5^**	**4.6×10^-5^**	0.0048
Natural killer cell	NKT.4+.Lv	**3.5×10^-5^**	**1.1×10^-4^**	0.019
T cell	LN.TR.14w.B6	**4.4×10^-5^**	**3.6×10^-5^**	7.4×10^-4^
T cell	ABD.TR.14w.B6	**5.4×10^-5^**	**6.5×10^-5^**	2.2×10^-4^
T cell	T.4Mem44h62l.LN	**1.4×10^-4^**	5.6×10^-4^	0.0078
T cell	CD4Control	3.3×10^-4^	**9.1×10^-5^**	0.0013
T cell	T.8Mem.Sp	0.010	0.0064	**1.5×10^-4^**
T cell	T.8Eff.Tbet+.Sp.OT1.d6LisOVA	0.053	0.051	**1.5×10^-4^**

P values satisfying the threshold of 0.05/292 for Bonferroni multiple testing are shown in bold.

BA, bronchial asthma; BBJ, BioBank Japan; PO, pollinosis; UKB, UK Biobank.

### Pathway enrichment in the autoimmune and allergic diseases

To elucidate pathogenicity, we conducted pathway enrichment analysis of the autoimmune and allergic disease GWASs with 150 gene sets in the lower layers of ‘immune system’ in Reactome. In the BBJ and UKB data sets, allergic diseases were significantly enriched in multiple gene sets in the lower layers of ‘cytokine signalling’, including IL−4, 5 and 13 involved in type 2 inflammation and IL-1,6, and TNF involved in non-type 2 inflammation (figure 4B). In the lower layers of ‘innate immune system’, BA is significantly associated with C-type lectin receptors and Dectin1 signalling, which is involved in house dust mite-induced allergic airway inflammation. As observed in the cell-type enrichment analysis, we observed less significant enrichment of the pathways in the autoimmune diseases. Only RA in BBJ was significantly associated with NOD1/2 signalling.

### Pervasive effect of the multitrait-associated variants on additional autoimmune diseases

**Figure 5:**
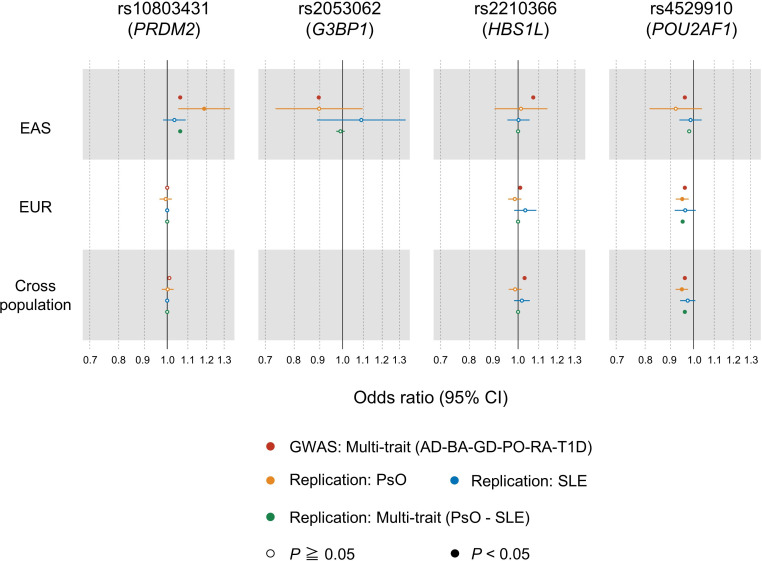
Forest plots of the replication meta-analysis for psoriasis and SLE. Odds ratio of the autoimmune and allergic associated variants are indicated by the individual population. The results of the original GWAS multi-trait analysis that integrates six autoimmune and allergic diseases are shown in red. The whiskers represent 95% CIs. AD, atopic dermatitis; BA, bronchial asthma; GD, Grave’s diseases; PO, pollinosis; PsO, psoriasis; RA, rheumatoid arthritis; SLE, systemic lupus erythematosus; T1D, type 1 diabetes.

We evaluated the effects of the four variants associated with autoimmune and allergic diseases on PsO and SLE by collecting additional individual data. Our replication meta-analysis included overall 21 778 cases and 712 767 controls in PsO and SLE (online supplemental table 2). We found nominally significant results consistent with our original multitrait GWAS meta-analysis for the two variants (rs10803431, OR=1.06, p=0.024 in EAS and rs4529910, OR=0.95, p=2.1×10^−4^ in EUR and OR=0.96, p=1.9×10^−4^ in cross-population; figure 5 and online supplemental table 3). The effect size of the EAS specific variant rs2053062 for PsO was similar to our multitrait analysis, while not significantly due to the limited sample size (OR=0.90, p=0.29 in EAS). From these results, our approach revealed the novel associations between genetic variants and additional autoimmune diseases.

### Drug targets for immune-related diseases at the identified multi-trait-associated loci

We found the biologically related genes in the allergic associated loci (68 in *CXCR4*, 19 in *CYP1B1*, and 8 in *ICOS*) and autoimmune and allergic associated loci (1 in *PRDM2*, 13 in *G3BP1*, 88 in *HBS1L*, 1 in *POU2AF1*) by using STRING V.11.5^40^ (online supplemental figure 11A). By querying them through DrugBank^41^ and TTD,^42^ we found that *CXCR4* and its functionally related genes have been therapeutic targets of various autoimmune and allergic diseases (online supplemental figure 11B). This result would be plausible given that chemokines involved in *CXCR4* broadly control the immune system.^43^ We also found that *ICOS* and its functionally related genes have been expected to be therapeutic targets of several autoimmune diseases. Given its ability to enhance T cell responses against foreign antigens,^44^ *ICOS* has the potential to be a common therapeutic target for autoimmune and allergic diseases.

## Discussion

In this study, the multitrait and cross-population GWAS meta-analysis depicted shared and distinct genetic components across the six immune-related diseases, which enabled de novo categorical classification of the autoimmune and allergic diseases solely based human genetics. Our study newly identified six loci associated with allergic diseases (*MIIP*, *CXCR4*, *SCML4*, *CYP1B1*, *ICOS* and *LINC00824*) and four pleiotropic loci associated with both autoimmune and allergic diseases (*PRDM2*, *G3BP1*, *HBS1L* and *POU2AF1*). While the variants identified in the meta-analysis in BBJ or UKB were ancestry specific (ie, almost monomorphic in the other ancestry), cross-population meta-analysis successfully enhanced the power to identify the variants with common effects between ancestry, thereby showing a value of both population-specific and cross-population approaches.

The European-specific *CYP1B1* missense variant of rs1800440 (N453S) was associated with allergic diseases susceptibility. Of note, another *CYP1B1* missense variant of rs1056836-G (V432L) was previously associated with BA susceptibility through a candidate gene approach (p=0.045),^45^ of which independent protective effect was also confirmed in our study (p=2.7×10^-5^). *CYP1B1* is a member of the cytochrome P450 superfamily of enzymes and performs ligand degradation in aryl hydrocarbon receptor (AHR)-dependent signalling pathway.^46^ AHR-dependent signalling pathway plays important roles in the immune response to molecular changes provided by the environment, diet, commensal flora and host metabolism.^47^ The missense variants of *CYP1B1* are involved in developing allergic diseases through the dysregulation of immune responses to external molecules.

The East Asian-specific putative causal variants in *G3BP1* were associated with autoimmune and allergic disease susceptibility. The lead variant of rs2053062 has been reported as an eQTL that affects *G3BP1* expression levels in multiple immune cells. Colocalisations between the eQTL and the GWAS data sets in a set of lymphocyte cell types suggested *G3BP1* as a potential risk gene in the loci. *G3BP1* plays a positive role in activating the STING pathway, resulting in type 1 interferon response.^48^ *G3BP1* expression levels have been reported to be high in autoimmune diseases involved in type 1 interferon, such as RA, myositis and SLE.^31^ Because rs2053062-T has been reported to decrease *G3BP1* expression levels, this variant may have a protective effect on disease susceptibility by suppressing type 1 interferon activation. Notably, the protective effect of rs2053062-T was also observed in the allergic diseases in our analysis, implying the involvement of type I interferon in allergy.

The cross-population meta-analysis identified the ancestry common variant in *POU2AF1*, which was associated with autoimmune and allergic disease susceptibility. The lead variant of *POU2AF1* also showed consistent effects on PsO and SLE. The *SIK2* locus, located downstream of *POU2AF1,* was previously reported to associate with allergic diseases. Several studies have annotated *POU2AF1* and *SIK2* together as the single risk locus. However, we found that these signals were independent. *POU2AF1* is essential for the response of B cells to antigens and required for the formation of germinal centres. *POU2AF1* is expressed in a highly cell-specific manner, being most abundant in B cells.^49^ The protective allele rs4529910-G has been reported to decrease *POU2AF1* expression levels in B cells.^31^ Therefore, we think that rs4529910-G has a protective effect for autoimmune and allergic diseases by attenuating humoral immunity.

Local heritability of allergic diseases was distributed across genome-wide, while it was relatively centralised in the HLA region in the autoimmune diseases. This difference might have resulted in heterogeneity in the enrichment analysis of cell type and biological pathway. In the cell-type enrichment analysis, regulatory T and natural killer cells were significantly enriched in allergic diseases, indicating the involvement of both adaptive and innate immune systems. The pathway enrichment analysis also showed that the allergic diseases were involved in various cytokine signals, including type 1 interferon. Non-type 2 inflammatory asthma is mainly caused by neutrophil inflammation involving IL6 and TNF-α, which is important pathogenesis as a cause of steroid refractory.^50^ Thus, our study captured the diverse aetiologies that compose the immune-related diseases.

We also acknowledge potential discussions. First, BBJ is a hospital-based cohort, while UKB is a population-based cohort. The difference in cohort characteristics, including prevalence and diagnosis criteria, may have affected the results. Second, the inclusion of the HLA region in estimating genome-wide heritability is challenging due to its complex genetic architecture. The general framework used in our analysis, LDSC, estimates polygenic effects without the HLA region. The relatively small polygenic effects in autoimmune diseases make several complex analyses more challenging (eg, cross-population genetic correlation^51^). Third, we reported the genetic loci satisfying the genome-wide significance threshold at the level of p=5.0×10^−8^ without multiple testing correction of the number of the GWAS. Recent multi-trait GWASs adopt the nominal genome-wide significance threshold of p=5.0×10^−8^.^3^ We note that the number of the significant loci was two (rs16902902 on *LINC00824* and rs4529910 on *POU2AF1*) when we strictly controlled multiple testing by Bonferroni correction (p<5.0×10^−8^/(12 independent GWASs and 9 meta-analyses)=*P*< 2.4×10^−9^).

In summary, our multi-trait and cross-population approaches utilising the large-scale biobank resources demonstrated evidence of both distinct and shared genetic components across the autoimmune and allergic diseases. We also provided identification of novel loci linked to the immune-related diseases as well as elucidation of disease pathogenicity. Our approach proposes novel strategies to understand genetic backgrounds, biology, therapeutic targets of a set of complex human traits such as immune-related diseases.

## Data Availability

Data are available upon reasonable request. The summary statistics of the GWAS results has been deposited in the National Bioscience Database Center (NBDC) Human Database (https://humandbs.biosciencedbc.jp/en/) under the accession number of hum0197 [https://humandbs.biosciencedbc.jp/en/hum0197-latest]. Data can also be browsed at our pheweb.jp website [https://pheweb.jp/].

## References

[1] Okada Y , Wu D , Trynka G , et al . Genetics of rheumatoid arthritis contributes to biology and drug discovery. Nature 2014;506:376–81. 10.1038/nature12873 24390342 PMC3944098

[2] Ferreira MA , Vonk JM , Baurecht H , et al . Shared genetic origin of asthma, hay fever and eczema elucidates allergic disease biology. Nat Genet 2017;49:1752–7. 10.1038/ng.3985 29083406 PMC5989923

[3] Sakaue S , Kanai M , Tanigawa Y , et al . A cross-population atlas of genetic associations for 220 human phenotypes. Nat Genet 2021;53:1415–24. 10.1038/s41588-021-00931-x 34594039 PMC12208603

[4] Li YR , Li J , Zhao SD , Bradfield JP , et al . Meta-Analysis of shared genetic architecture across ten pediatric autoimmune diseases. Nat Med 2015;21:1018–27. 10.1038/nm.3933 26301688 PMC4863040

[5] Márquez A , Kerick M , Zhernakova A , et al . Meta-Analysis of immunochip data of four autoimmune diseases reveals novel Single-Disease and cross-phenotype associations. Genome Med 2018;10:1–13. 10.1186/s13073-018-0604-8 30572963 PMC6302306

[6] Acosta-Herrera M , Kerick M , González-Serna D , et al . Genome-wide meta-analysis reveals shared new loci in systemic seropositive rheumatic diseases. Ann Rheum Dis 2019;78:311–9. 10.1136/annrheumdis-2018-214127 30573655 PMC6800208

[7] Rottem M , Gershwin ME , Shoenfeld Y . Allergic disease and autoimmune effectors pathways. Dev Immunol 2002;9:161–7. 10.1080/1044667031000137638 12885156 PMC2276104

[8] Krishna MT , Subramanian A , Adderley NJ , et al . Allergic diseases and long-term risk of autoimmune disorders: longitudinal cohort study and cluster analysis. Eur Respir J 2019;54. 10.1183/13993003.00476-2019. [Epub ahead of print: 14 11 2019]. 31413164

[9] Nagai A , Hirata M , Kamatani Y , et al . Overview of the Biobank Japan project: study design and profile. J Epidemiol 2017;27:S2–8. 10.1016/j.je.2016.12.005 28189464 PMC5350590

[10] Bycroft C , Freeman C , Petkova D , et al . The UK Biobank resource with deep phenotyping and genomic data. Nature 2018;562:203–9. 10.1038/s41586-018-0579-z 30305743 PMC6786975

[11] Hirata M , Kamatani Y , Nagai A , et al . Cross-Sectional analysis of Biobank Japan clinical data: a large cohort of 200,000 patients with 47 common diseases. J Epidemiol 2017;27:S9–21. 10.1016/j.je.2016.12.003 28190657 PMC5363792

[12] Sudlow C , Gallacher J , Allen N , et al . Uk Biobank: an open access resource for identifying the causes of a wide range of complex diseases of middle and old age. PLoS Med 2015;12:e1001779–10. 10.1371/journal.pmed.1001779 25826379 PMC4380465

[13] Hirata J , Hosomichi K , Sakaue S , et al . Genetic and phenotypic landscape of the major histocompatibilty complex region in the Japanese population. Nat Genet 2019;51:470–80. 10.1038/s41588-018-0336-0 30692682

[14] Akiyama M , Ishigaki K , Sakaue S , et al . Characterizing rare and low-frequency height-associated variants in the Japanese population. Nat Commun 2019;10:4393. 10.1038/s41467-019-12276-5 31562340 PMC6764965

[15] Okada Y , Momozawa Y , Sakaue S , et al . Deep whole-genome sequencing reveals recent selection signatures linked to evolution and disease risk of Japanese. Nat Commun 2018;9:1631. 10.1038/s41467-018-03274-0 29691385 PMC5915442

[16] Zhou W , Nielsen JB , Fritsche LG , et al . Efficiently controlling for case-control imbalance and sample relatedness in large-scale genetic association studies. Nat Genet 2018;50:1335–41. 10.1038/s41588-018-0184-y 30104761 PMC6119127

[17] Bulik-Sullivan BK , Loh P-R , Finucane HK , et al . LD score regression distinguishes confounding from polygenicity in genome-wide association studies. Nat Genet 2015;47:291–5. 10.1038/ng.3211 25642630 PMC4495769

[18] Ning Z , Pawitan Y , Shen X . High-Definition likelihood inference of genetic correlations across human complex traits. Nat Genet 2020:1–6. 32601477 10.1038/s41588-020-0653-y

[19] Zhang Y , Lu Q , Ye Y , et al . SUPERGNOVA: local genetic correlation analysis reveals heterogeneous etiologic sharing of complex traits. Genome Biol 2021;22:1–30. 10.1186/s13059-021-02478-w 34493297 PMC8422619

[20] Berisa T , Pickrell JK . Approximately independent linkage disequilibrium blocks in human populations. Bioinformatics 2016;32:btv546. 10.1093/bioinformatics/btv546 PMC4731402 26395773

[21] Lin D-Y , Sullivan PF . Meta-Analysis of genome-wide association studies with overlapping subjects. Am J Hum Genet 2009;85:862–72. 10.1016/j.ajhg.2009.11.001 20004761 PMC2790578

[22] Lee CH , Eskin E , Han B . Increasing the power of meta-analysis of genome-wide association studies to detect heterogeneous effects. Bioinformatics 2017;33:i379–88. 10.1093/bioinformatics/btx242 28881976 PMC5870848

[23] Watanabe K , Taskesen E , van Bochoven A , et al . Functional mapping and annotation of genetic associations with FUMA. Nat Commun 2017;8:1–10. 10.1038/s41467-017-01261-5 29184056 PMC5705698

[24] Buniello A , MacArthur JAL , Cerezo M , et al . The NHGRI-EBI GWAS catalog of published genome-wide association studies, targeted arrays and summary statistics 2019. Nucleic Acids Res 2019;47:D1005–12. 10.1093/nar/gky1120 30445434 PMC6323933

[25] Gagliano Taliun SA , VandeHaar P , Boughton AP , et al . Exploring and visualizing large-scale genetic associations by using PheWeb. Nat Genet 2020;52:550–2. 10.1038/s41588-020-0622-5 32504056 PMC7754083

[26] Kamat MA , Blackshaw JA , Young R , et al . PhenoScanner V2: an expanded tool for searching human genotype-phenotype associations. Bioinformatics 2019;35:4851–3. 10.1093/bioinformatics/btz469 31233103 PMC6853652

[27] Ghoussaini M , Mountjoy E , Carmona M , et al . Open targets genetics: systematic identification of trait-associated genes using large-scale genetics and functional genomics. Nucleic Acids Res 2021;49:D1311–20. 10.1093/nar/gkaa840 33045747 PMC7778936

[28] Wang G , Sarkar A , Carbonetto P , et al . A simple new approach to variable selection in regression, with application to genetic fine mapping. J R Stat Soc B 2020;82:1273–300. 10.1111/rssb.12388 PMC10201948 37220626

[29] Wang K , Li M , Hakonarson H . ANNOVAR: functional annotation of genetic variants from high-throughput sequencing data. Nucleic Acids Res 2010;38:e164–7. 10.1093/nar/gkq603 20601685 PMC2938201

[30] GTEx Consortium . The GTEx Consortium atlas of genetic regulatory effects across human tissues. Science 2020;369:1318–30. 10.1126/science.aaz1776 32913098 PMC7737656

[31] Ota M , Nagafuchi Y , Hatano H , et al . Dynamic landscape of immune cell-specific gene regulation in immune-mediated diseases. Cell 2021;184:3006–21. 10.1016/j.cell.2021.03.056 33930287

[32] Hormozdiari F , van de Bunt M , Segrè AV , et al . Colocalization of GWAS and eQTL signals detects target genes. Am J Hum Genet 2016;99:1245–60. 10.1016/j.ajhg.2016.10.003 27866706 PMC5142122

[33] Finucane HK , Reshef YA , Anttila V , et al . Heritability enrichment of specifically expressed genes identifies disease-relevant tissues and cell types. Nat Genet 2018;50:621–9. 10.1038/s41588-018-0081-4 29632380 PMC5896795

[34] Heng TSP , Painter MW , Elpek K , Immunological Genome Project Consortium . The immunological genome Project: networks of gene expression in immune cells. Nat Immunol 2008;9:1091–4. 10.1038/ni1008-1091 18800157

[35] Lamparter D , Marbach D , Rueedi R , et al . Fast and rigorous computation of gene and pathway scores from SNP-based summary statistics. PLoS Comput Biol 2016;12:e1004714–20. 10.1371/journal.pcbi.1004714 26808494 PMC4726509

[36] Subramanian A , Tamayo P , Mootha VK , et al . Gene set enrichment analysis: a knowledge-based approach for interpreting genome-wide expression profiles. Proc Natl Acad Sci U S A 2005;102:15545–50. 10.1073/pnas.0506580102 16199517 PMC1239896

[37] Shannon P , Markiel A , Ozier O , et al . Cytoscape: a software environment for integrated models of biomolecular interaction networks. Genome Res 2003;13:2498–504. 10.1101/gr.1239303 14597658 PMC403769

[38] Wang Y-F , Zhang Y , Lin Z , et al . Identification of 38 novel loci for systemic lupus erythematosus and genetic heterogeneity between ancestral groups. Nat Commun 2021;12:1–13. 10.1038/s41467-021-21049-y 33536424 PMC7858632

[39] Bentham J , Morris DL , Graham DSC , et al . Genetic association analyses implicate aberrant regulation of innate and adaptive immunity genes in the pathogenesis of systemic lupus erythematosus. Nat Genet 2015;47:1457–64. 10.1038/ng.3434 26502338 PMC4668589

[40] Szklarczyk D , Gable AL , Lyon D , et al . String v11: protein-protein association networks with increased coverage, supporting functional discovery in genome-wide experimental datasets. Nucleic Acids Res 2019;47:D607–13. 10.1093/nar/gky1131 30476243 PMC6323986

[41] Wishart DS , Feunang YD , Guo AC , et al . DrugBank 5.0: a major update to the DrugBank database for 2018. Nucleic Acids Res 2018;46:D1074–82. 10.1093/nar/gkx1037 29126136 PMC5753335

[42] Zhou Y , Zhang Y , Lian X , et al . Therapeutic target database update 2022: facilitating drug discovery with enriched comparative data of targeted agents. Nucleic Acids Res 2022;50:D1398–407. 10.1093/nar/gkab953 34718717 PMC8728281

[43] García-Cuesta EM , Santiago CA , Vallejo-Díaz J , et al . The role of the CXCL12/CXCR4/ACKR3 axis in autoimmune diseases. Front Endocrinol 2019;10:1–16. 10.3389/fendo.2019.00585 PMC6718456 31507535

[44] Hutloff A , Dittrich AM , Beier KC , et al . ICOS is an inducible T-cell co-stimulator structurally and functionally related to CD28. Nature 1999;397:263–6. 10.1038/16717 9930702

[45] Polonikov AV , Ivanov VP , Solodilova MA . Genetic variation of genes for xenobiotic-metabolizing enzymes and risk of bronchial asthma: the importance of gene-gene and gene-environment interactions for disease susceptibility. J Hum Genet 2009;54:440–9. 10.1038/jhg.2009.58 19575027

[46] Quintana FJ . Review regulation of the immune response by the aryl hydrocarbon receptor, 2018: 19–33. 10.1016/j.immuni.2017.12.012 PMC5777317 29343438

[47] Rothhammer V , Quintana FJ . The aryl hydrocarbon receptor: an environmental sensor integrating immune responses in health and disease. Nat Rev Immunol 2019;19:184–97. 10.1038/s41577-019-0125-8 30718831

[48] Wiser C , Kim B , Ascano M . G3Bp1 enhances cytoplasmic DNA pattern recognition. Nat Immunol 2019;20:5–7. 10.1038/s41590-018-0279-8 30538338

[49] Strubin M , Newell JW , Matthias P . Obf-1, a novel B cell-specific coactivator that stimulates immunoglobulin promoter activity through association with octamer-binding proteins. Cell 1995;80:497–506. 10.1016/0092-8674(95)90500-6 7859290

[50] Israel E , Reddel HK , Severe RHK . Severe and difficult-to-treat asthma in adults. N Engl J Med 2017;377:965–76. 10.1056/NEJMra1608969 28877019

[51] Galinsky KJ , Reshef YA , Finucane HK , et al . Estimating cross-population genetic correlations of causal effect sizes. Genet Epidemiol 2019;43:180–8. 10.1002/gepi.22173 30474154 PMC6375794

