# Supplementary material for: Multi-trait and cross-population genome-wide association studies across autoimmune and allergic diseases identify shared and distinct genetic component
Source: Ann Rheum Dis. 2022 Jun 26;81(9):1301–12. doi: 10.1136/annrheumdis-2022-222460 (PMC9380494; doi:10.1136/annrheumdis-2022-222460)
Supplement: Supplementary data [file annrheumdis-2022-222460supp001.pdf]

Multi-trait and cross-population genome-wide association studies integrating autoimmune and allergic diseases identifies shared genetic components.

Yuya Shirai et al.

Corresponding to Prof. Yukinori Okada (yokada@sg.med.osaka-u.ac.jp).

Supplementary Figure 1. Local heritability in the autoimmune and allergic diseases.

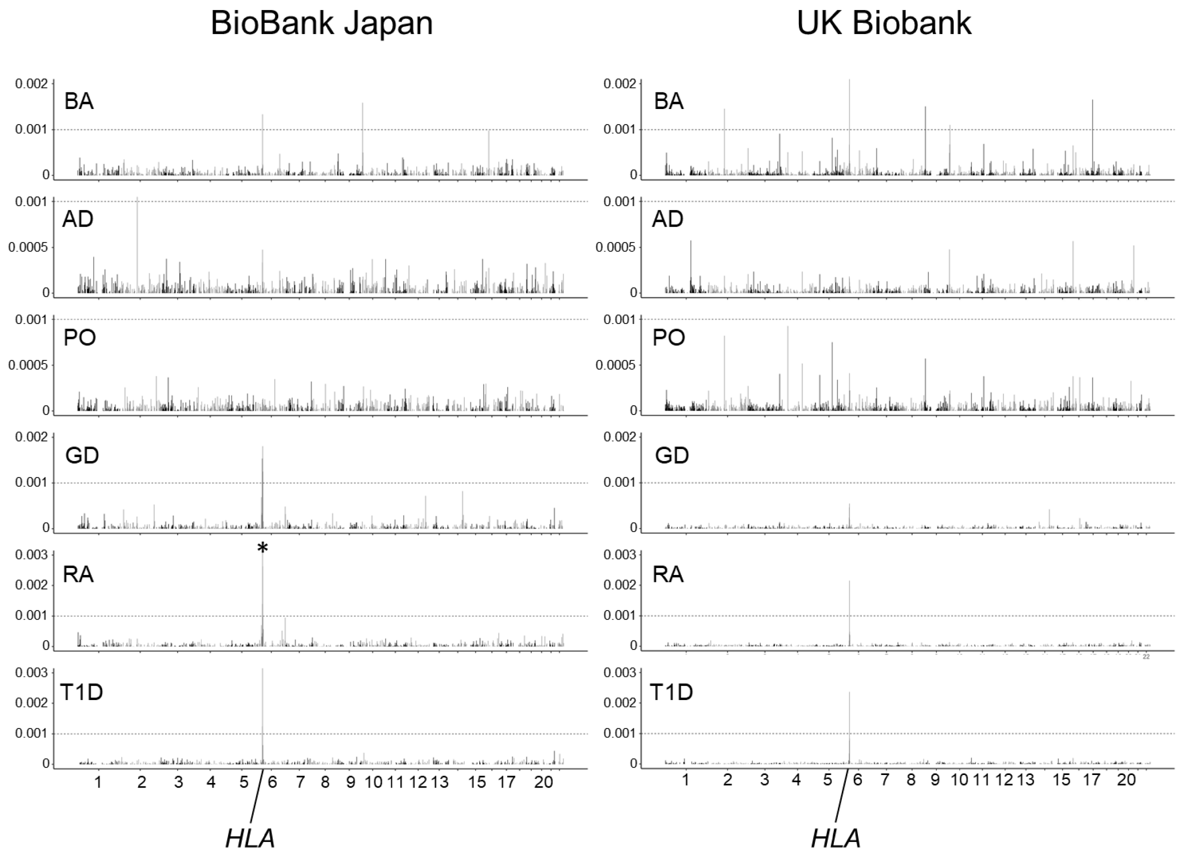

The local heritability is depicted along the genomic positions. The local heritability was calculated per LD-independent segment (1,442 segments in BBJ and 1,700 segments in UKB). The horizontal dashed black line indicates local heritability of 0.01. The asterisk in BBJ RA indicates that the local heritability exceeds the upper limit of the figure, which was estimated to be 0.0096. AD, atopic dermatitis; BA, bronchial asthma; GD, Grave's diseases; PO, pollinosis; RA, rheumatoid arthritis; T1D, type 1 diabetes.

Supplementary Figure 2. Cumulative percentage of local heritability in the autoimmune and allergic diseases.

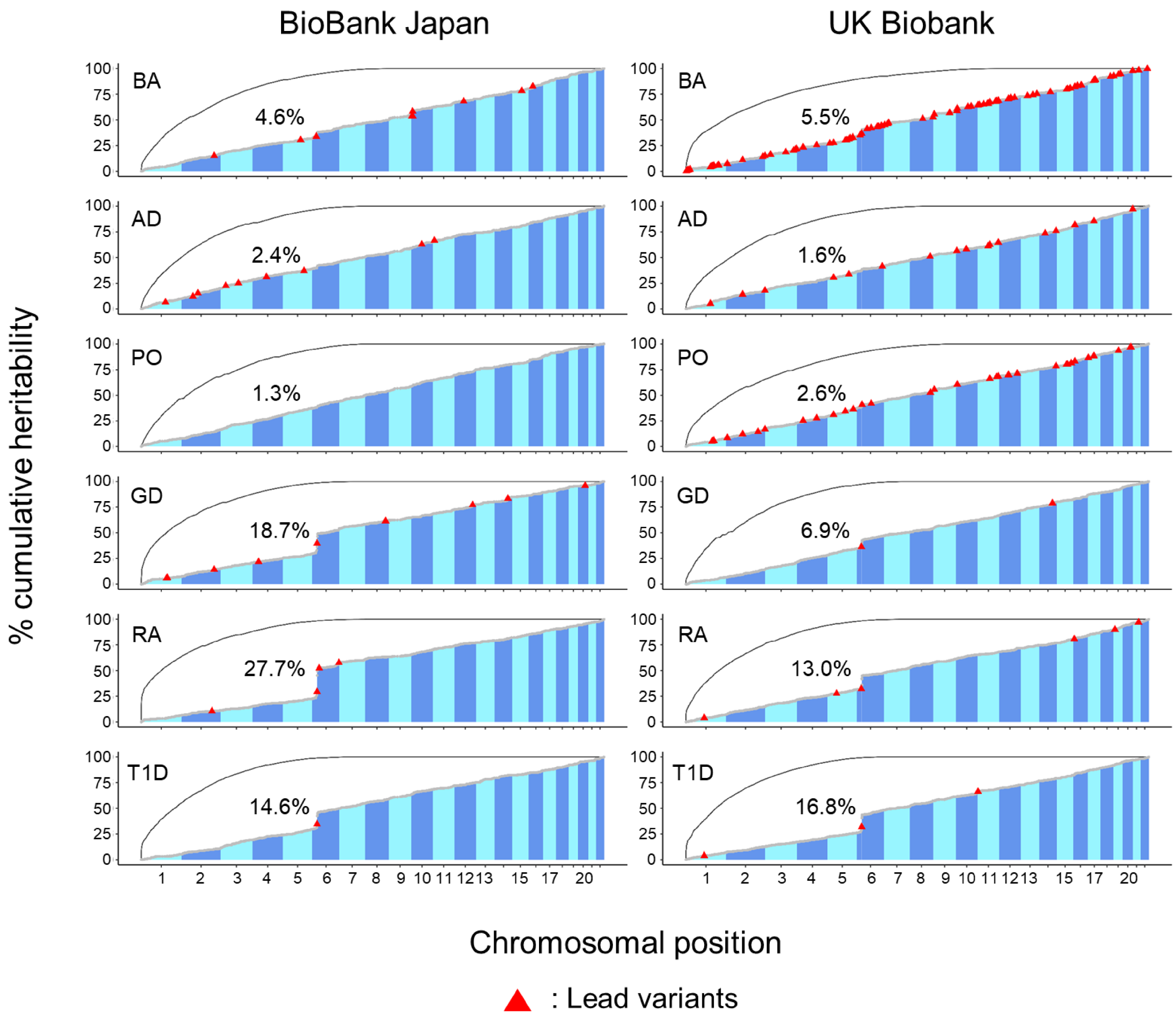

The gray dots indicate the cumulative percentage of local heritability for each LD block, highlighted in red triangles when the LD block contained GWAS lead variants. The black lines show the cumulative curves of local heritability totaled in descending order. The percentages of local heritability contributed by the HLA region (chr6:26Mb-34Mb) are shown in the figure. AD, atopic dermatitis; BA, bronchial asthma; GD, Grave's diseases; PO, pollinosis; RA, rheumatoid arthritis; T1D, type 1 diabetes.

Supplementary Figure 3. Local genetic correlation among the autoimmune and allergic diseases.

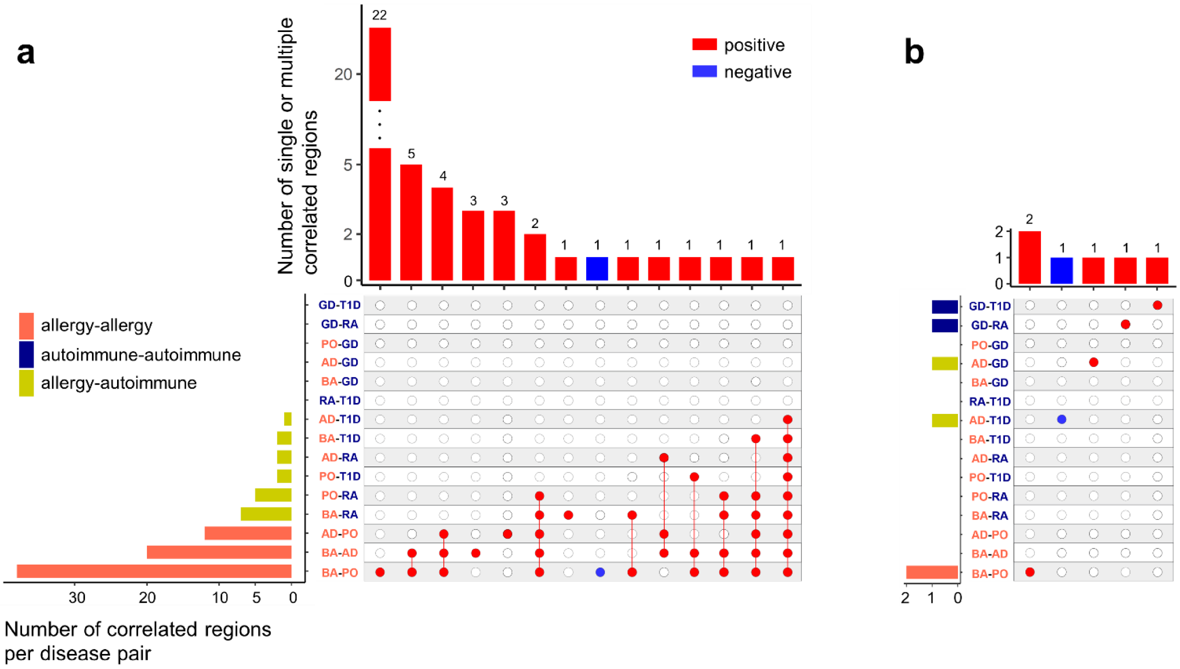

(**a**) In the UKB GWAS, a region with significant positive genetic correlation is represented as a red node, and a region with significant negative genetic correlation is represented as a blue node. The significance threshold was adopted at the level of *FDR* = 0.05. The upper histograms describe the number of single or multiple correlated regions. The left histograms describe the number of significant regions per disease pair, which are colored according to disease categories. (**b**) In the BBJ GWAS, correlated regions are described in the same format as (a). AD, atopic dermatitis; BA, bronchial asthma; GD, Grave's diseases; PO, pollinosis; RA, rheumatoid arthritis; T1D, type 1 diabetes.

Supplementary Figure 4. A hub region where multiple immune-related diseases correlated.

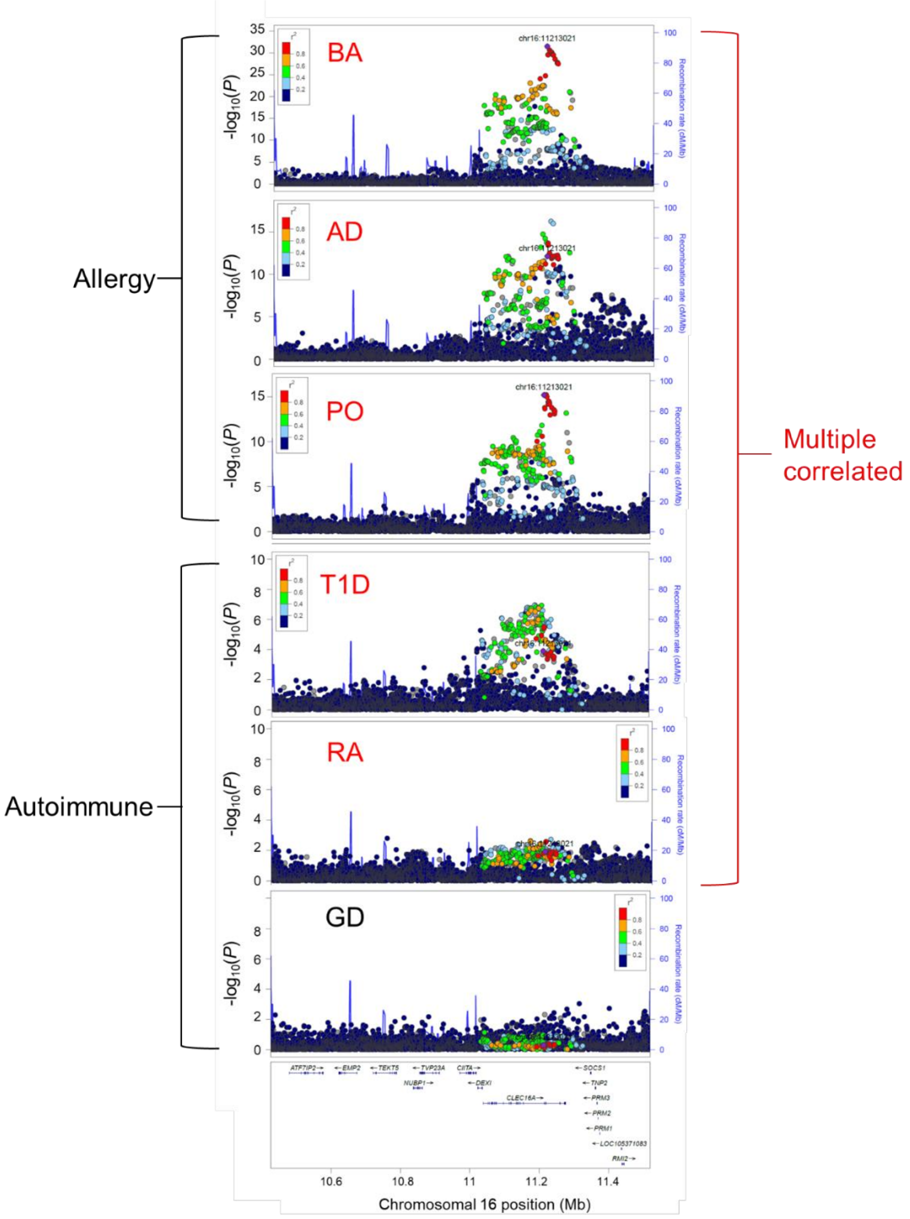

Regional plots for the locus where nine pairs were positively correlated in the UKB GWAS. The nine pairs were formed between the diseases colored in red except for T1D-RA. The lead variants in the BA GWAS are colored in purple and all the other variants are colored based on LD with the lead variant as in the legend. AD, atopic dermatitis; BA, bronchial asthma; GD, Grave's diseases; PO, pollinosis; RA, rheumatoid arthritis; T1D, type 1 diabetes.

Supplementary Figure 5. Quantile–quantile (QQ) plots of GWAS meta-analysis.

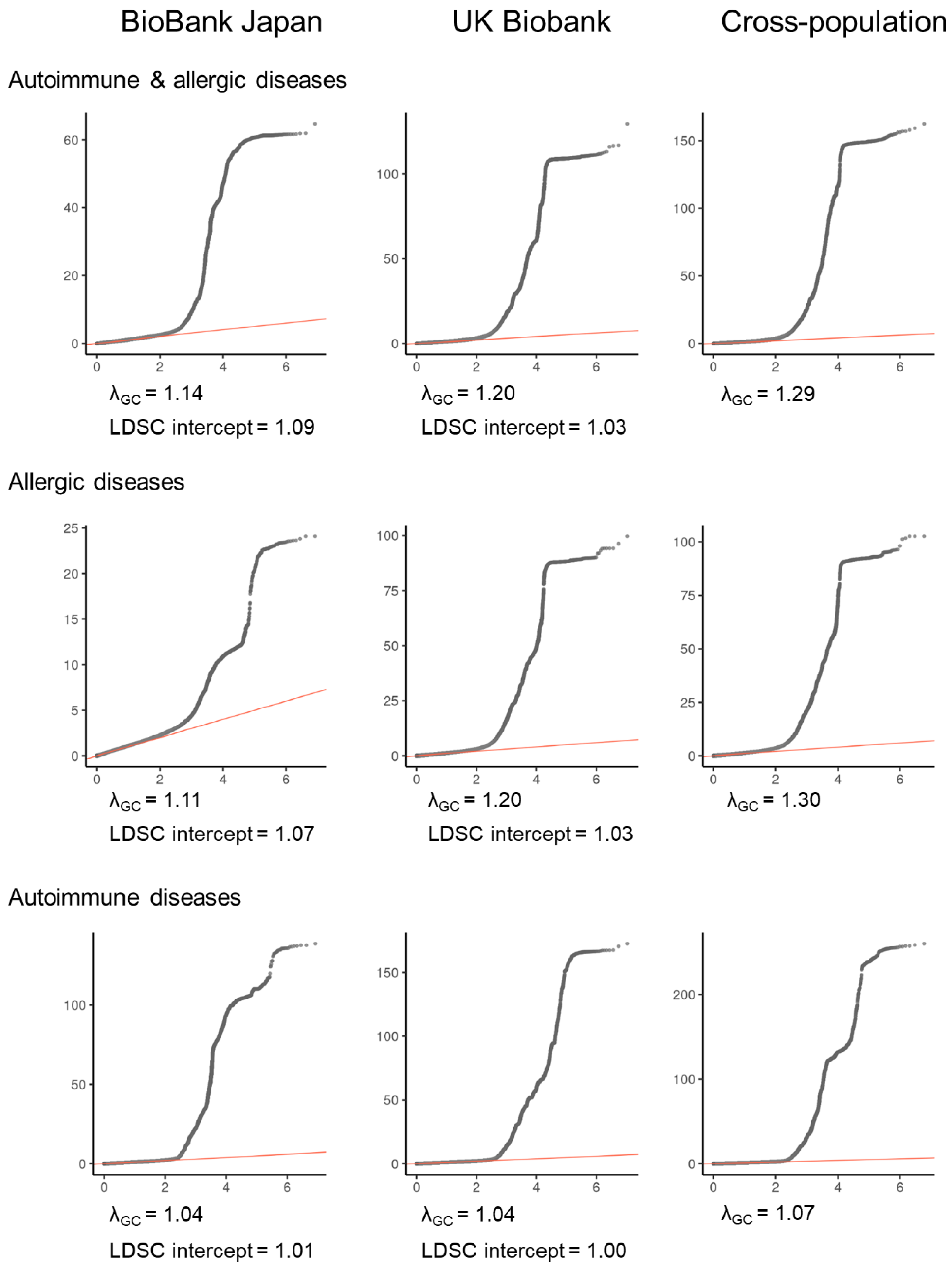

The x-axis indicates the expected -$\log_{10}$(*P*-values) and the y-axis indicates the observed -$\log_{10}$(*P*-values). The genomic inflation factor (λ_{GC}) and intercept estimated by LD score regression in BBJ and UKB cohorts are shown under the individual figure.

Supplementary Figure 6. Statistical fine-mapping of population specific and common disease associated loci.

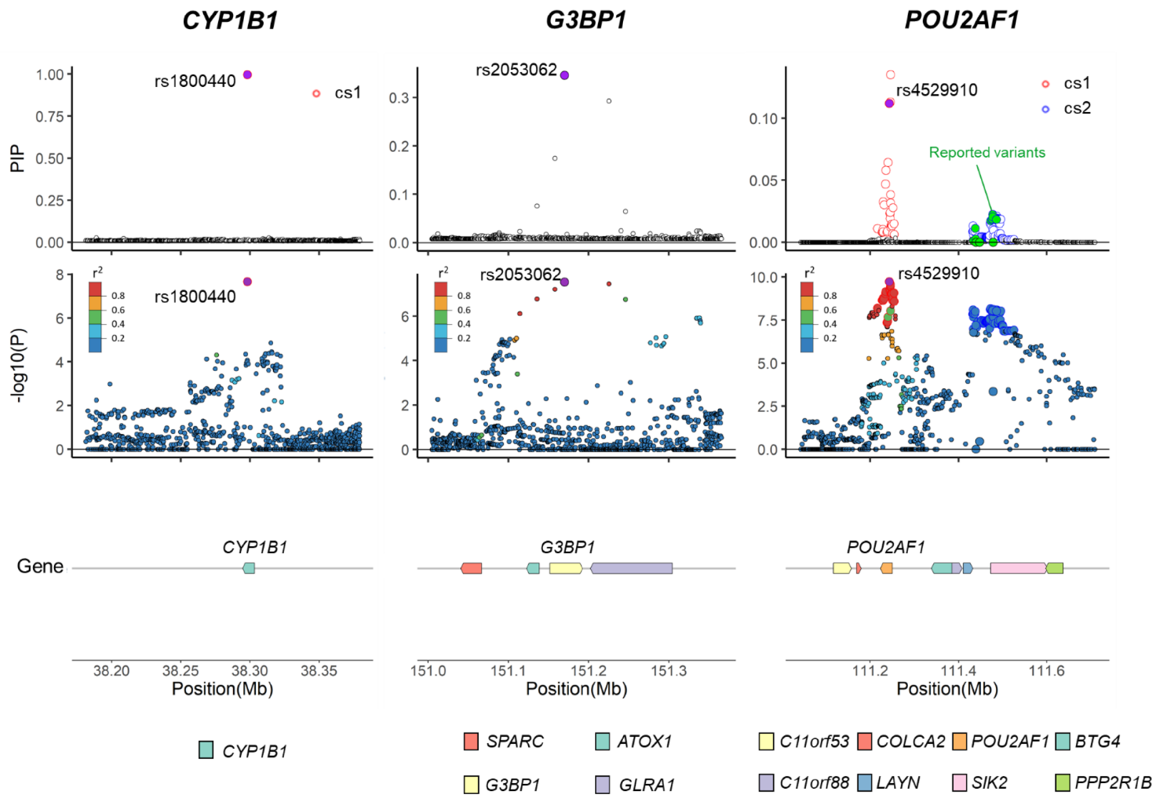

The posterior inclusion probabilities (PIP) calculated by SuSiE are pointed in the UKB specific locus (left), the BBJ specific locus (middle), and the ancestry common locus (right), along with the region plot. The individual lead variants are shown in the plot. CS, 95% credible set.

Supplementary Figure 7. Heatmaps describing the significance of individual GWAS.

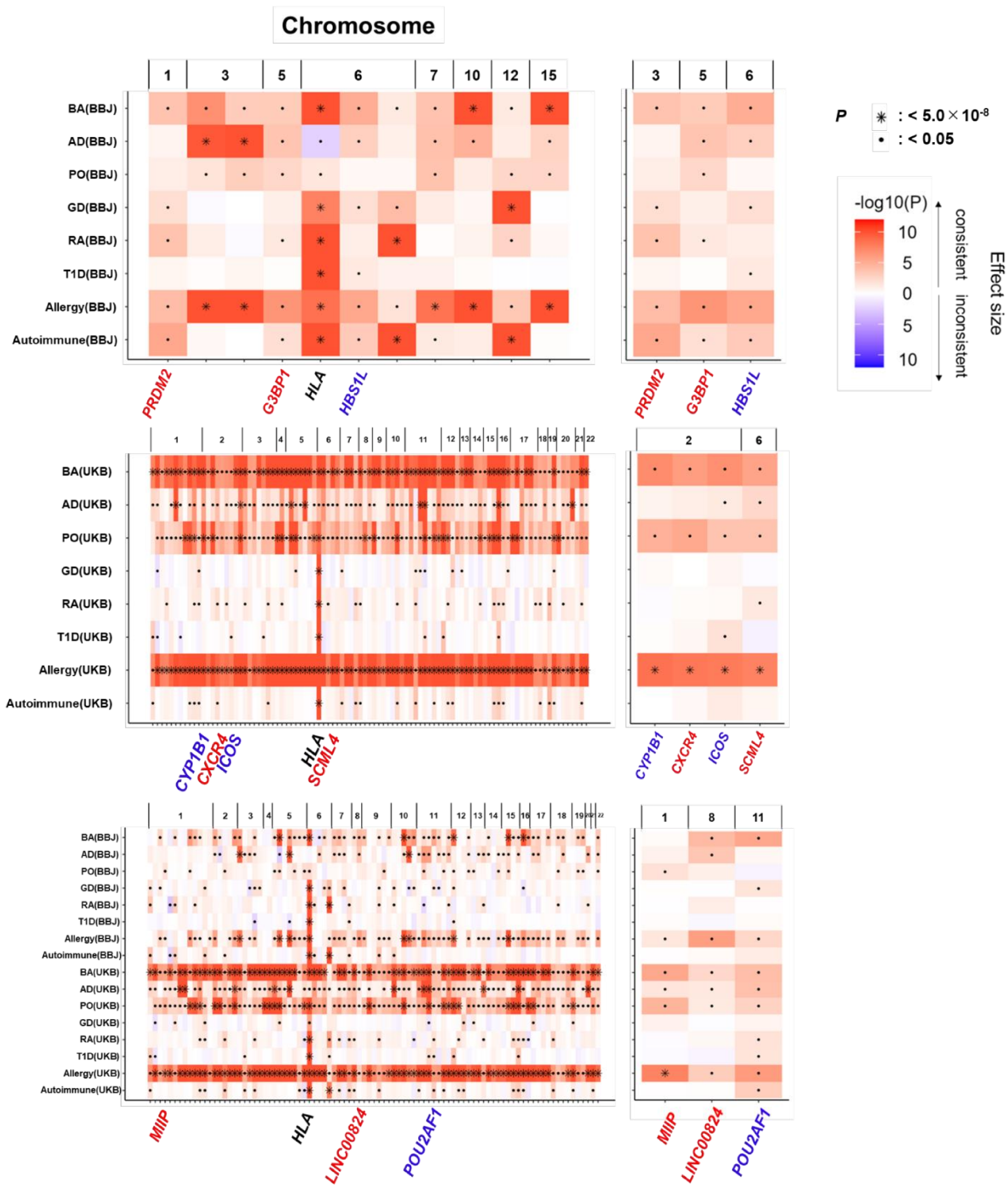

As for the genome-wide significant ($P < 5.0 \times 10^{-8}$) variants in the autoimmune and allergic meta-analyses, -log10(P) in individual GWAS is represented in the heat map along the genome position: upper, BBJ; middle, UKB; lower, cross-population. If the direction of the effect is consistent with the meta-analysis, the cell is colored in red, otherwise colored in blue. The novel loci (red) and independent loci (blue) identified in the meta-analysis and *HLA* are shown.

Supplementary Figure 8. eQTL effect of rs12702634 on *G3BP1* from ImmuNexUT.

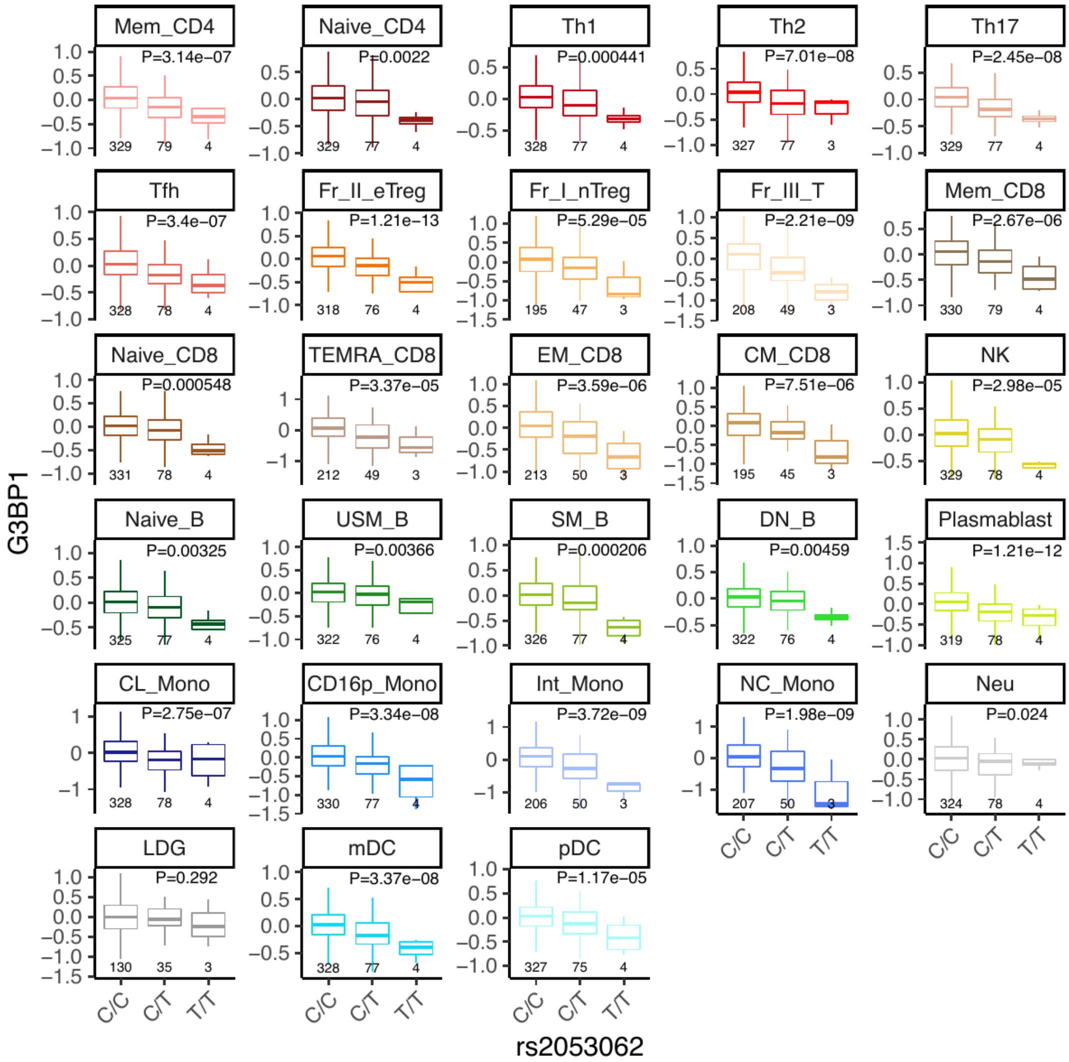

Box plots of *G3BP1* expression levels in 28 immune cells by genotype. The x-axis indicates the genotype, where the reference allele is C and the alternate is T. The numbers below the individual box plots indicate sample size. The y-axis indicates the normalized gene expression level of *G3BP1*.

Supplementary Figure 9. Colocalization analysis in *G3BP1* locus.

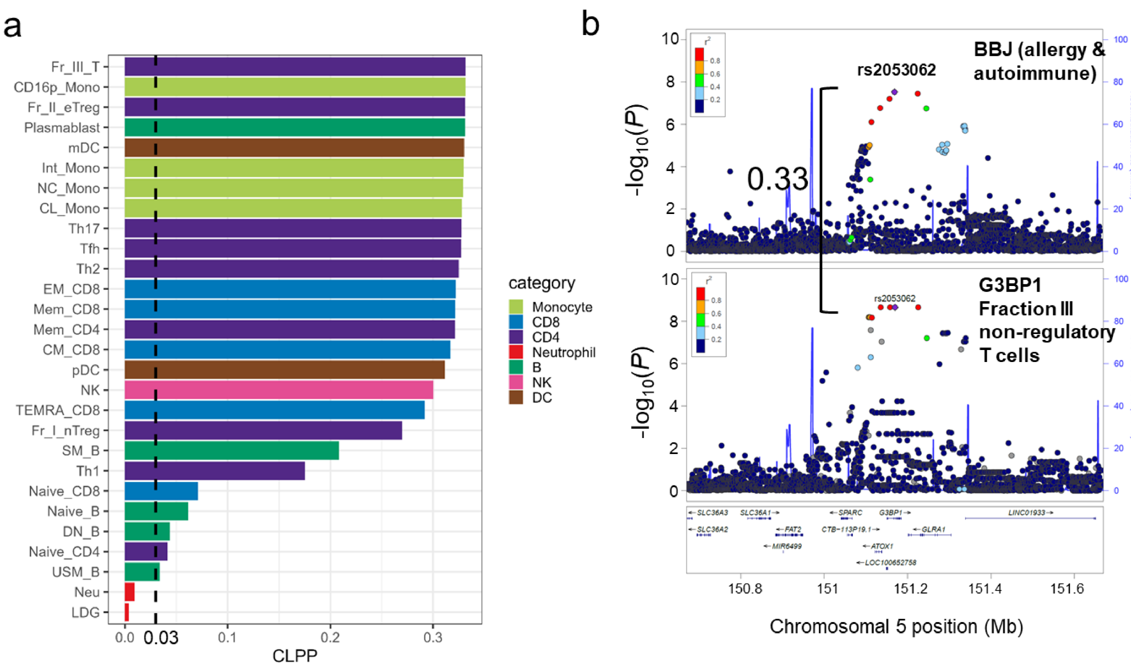

(**a**) The colocalization posterior probability (CLPP) calculated by eCAVIAR is represented in the histogram. In colocalization analysis, Z score in the BBJ meta-analysis and Z score of eQTL data in ImmuNexUT are compared. The horizontal dashed line indicates the colocalization significance threshold of 0.03. (**b**) The region plots of the locus suggesting the most significant colocalization (CLPP = 0.33).

Supplementary Figure 10. eQTL effect of rs4529910 on *POU2AF1* from ImmuNexUT.

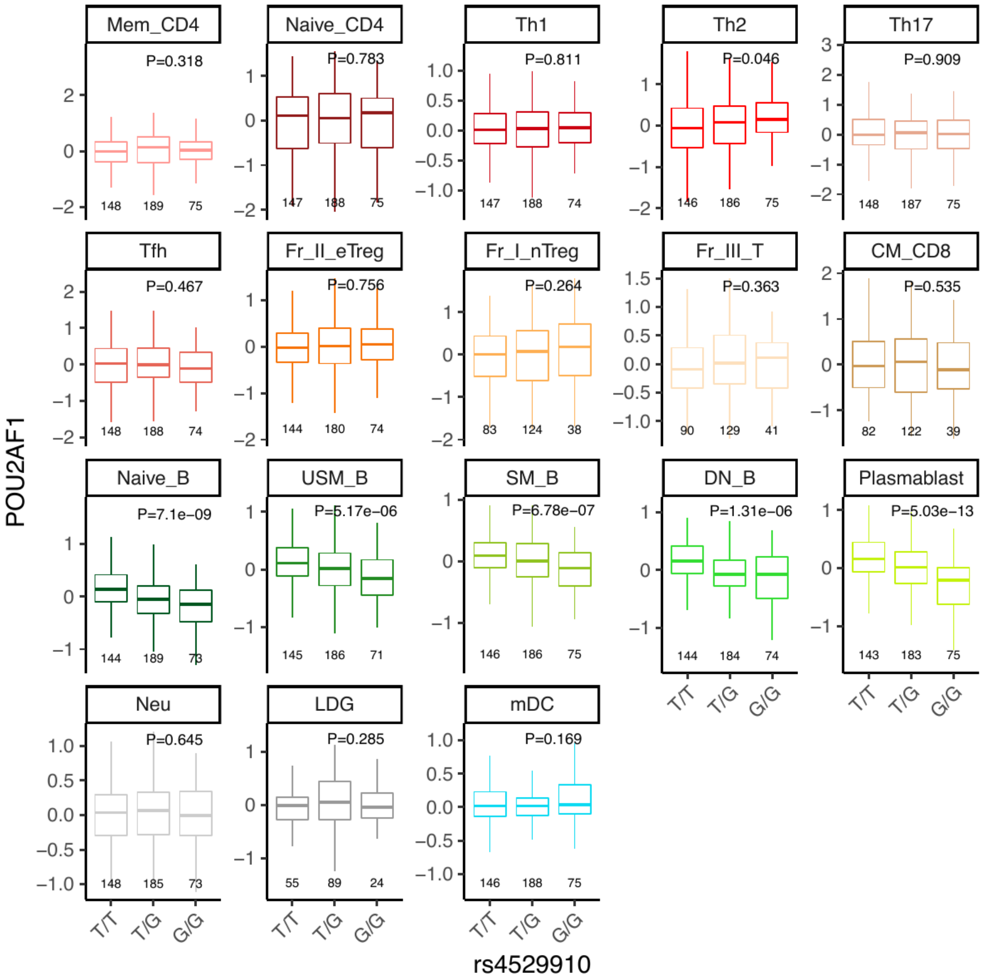

Box plots of *POU2AF1* expression levels in 18 immune cells by genotype. The x-axis indicates the genotype, where the reference allele is T and the alternate is G. The numbers below the individual box plots indicate sample size. The y-axis indicates the normalized gene expression level of *POU2AF1*.

Supplementary Figure 11. Therapeutic targets for the genes associated with autoimmune and allergic diseases.

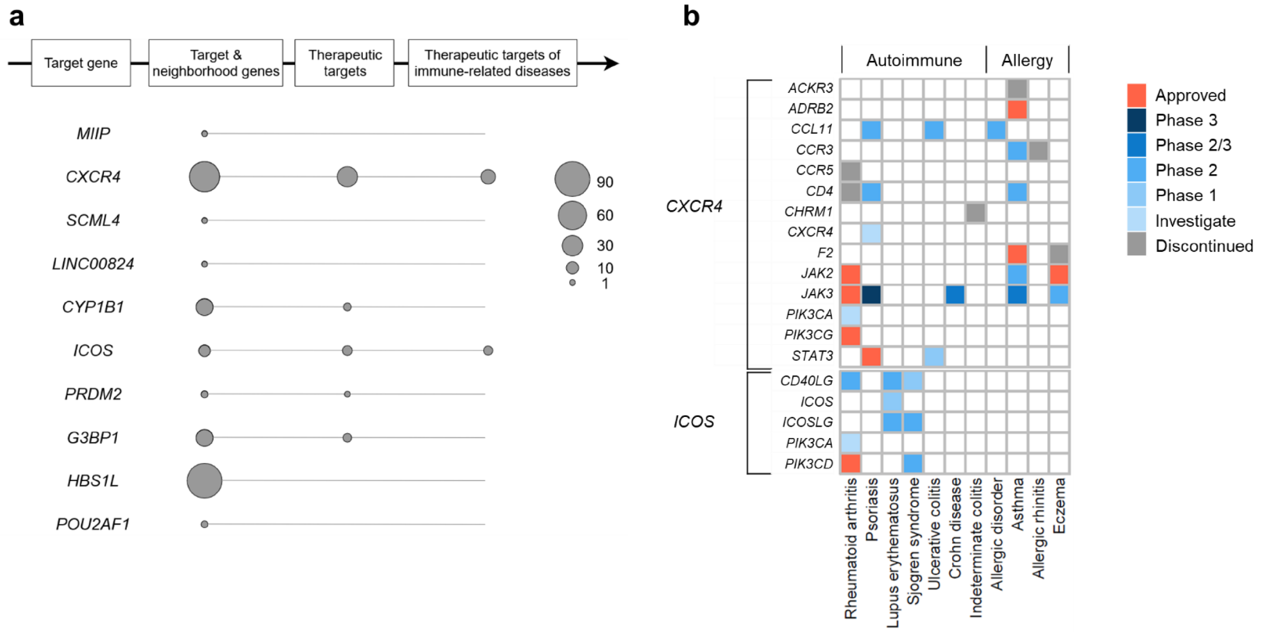

(**a**) Balloon plot of the number of target and neighborhood genes. Target genes are 10 genes we identified in this study. Neighborhood genes are defined by protein-protein interaction (PPI) score with the target genes in STRING V.11.5 (combined score excluding “text mining score” > 0.7). Therapeutic targets are genes registered in Therapeutic Target Database (TTD). (b) Heat map showing the stages for clinical use concerning therapeutic targets of immune-related diseases. The referenced TTD data was last updated on September 29th, 2021.

Supplementary Table 1. Summary of study subjects and individual-trait genome-wide association study (GWAS)

Phenotype		BBJ											UKB										
		Case			Control			Case freq	GWAS				Case			Control			Case freq	GWAS			
		No.	Age mean ±SD	Male No. (%)	No.	Age mean ±SD	Male No. (%)		Sig.loci*	λ_{GC}	Heritability	LDSC intercept	No.	Age mean ±SD	Male No. (%)	No.	Age mean ±SD	Male No. (%)		Sig.loci*	λ_{GC}	Heritability	LDSC intercept
Allergic diseases	Graves' disease(GD)	2,041	50.1 ± 15.3	569 (0.28)	142,192	64.1 ± 13.3	79,317 (0.56)	0.014	9	1.017	0.017	0.981	614	55.9 ± 8.0	110 (0.18)	291,471	57.0 ± 8.0	155,033 (0.47)	0.0021	2	1.029	0.0021	1.004
	Rheumatoid arthritis(RA)	2,370	61.9 ± 12.0	457 (0.19)				0.016	4	1.017	0.021	0.976	5,065	59.3 ± 7.1	1,586 (0.31)				0.017	6	1.026	0.0083	0.989
	Type 1 DM(T1D)	638	51.0 ± 16.7	324 (0.51)				0.0045	1	1.029	0.0037	1.006	914	54.9 ± 8.1	489 (0.54)				0.003	3	1.035	0.0018	1.017
Autoimmune diseases	Asthma (BA)	7,522	56.2 ± 18.6	3,649 (0.49)				0.050	8	1.055	0.029	1.005	54,872	56.5 ± 8.2	23,340 (0.43)				0.158	88	1.189	0.063	1.016
	Atopic dermatitis(AD)	2,472	32.1 ± 12.9	1,327 (0.54)				0.017	9	1.037	0.015	1.002	12,285	55.5 ± 8.2	5,615 (0.46)				0.040	17	1.057	0.017	1.007
	Pollinosis (PO)	5,308	47.0 ± 16.2	2,290 (0.43)				0.036	0	1.028	0.0091	1.009	26,758	55.1 ± 8.1	12,538 (0.47)				0.084	34	1.121	0.035	1.007

* The loci satisfying the genome-wide significance threshold at the level of P = 5.0 × 10^{-8}.

Supplementary Table 2. Summary of the subjects in the replication meta-analysis

Population	East Asian						European					
Data type	Dosage data				Summary statistics		Dosage data		Summary statistics			
Cohort	BBJ		Osaka University		YF Wang et al. *Nat Commun* (2021)		UKB		FinnGen		J Bentham et al. *Nat Genet* (2015)	
	Case	Control	Case	Control	Case	Control	Case	Control	Case	Control	Case	Control
PsO	223	142,009	353	50,569	-	-	6,721	291,471	4,510	212,242	-	-
SLE	343	141,935	220	50,569	4,222	8,431	628	291,471	538	213,145	4,036	6,959
Multi-trait (PsO-SLE)	566	142,192	573	50,569	4,222	8,431	7,333	291,471	5,048	213,145	4,036	6,959

PsO, psoriasis; SLE, systemic lupus erythematosus
The sample size for multi-trait is the union of the individual dataset adjusted for sample overlap.

Shirai Y, *et al. Ann Rheum Dis* 2022; 81:1301–1312. doi: 10.1136/annrheumdis-2022-222460

Supplementary Table 3. Summary of the replication meta-analysis for psoriasis and SLE

SNP	Trait	East Asian				European				Cross population			
		Case	Control	OR	*P* †	Case	Control	OR	*P* †	Case	Control	OR	*P* †
rs10803431 (*PRDM2*)	GWAS: Multi-trait (AD-BA-GD-PO-RA-T1D)	19,609	142,192	1.06	$2.3{\times}10^{-8}$	86,112	291,471	1.00	0.42	105,721	433,663	1.01	0.058
	Replication: PsO	576	192,578	1.19	**0.0053**	11,231	503,713	0.99	0.62	11,807	696,291	1.00	0.87
	Replication: SLE	4,785	200,935	1.03	0.21	5,202	511,575	1.00	1.00	9,987	712,510	1.00	1.00
	Replication: Multi-trait (PsO-SLE)	5,361	201,192	1.06	**0.024**	16,417	511,575	1.00	1.00	21,778	712,767	1.00	1.00
rs2053062 (*G3BP1*)	GWAS: Multi-trait (AD-BA-GD-PO-RA-T1D)	19,609	142,192	0.90	$2.9{\times}10^{-8}$	-	-	-	-	-	-	-	-
	Replication: PsO	576	192,578	0.90	0.29	-	-	-	-	-	-	-	-
	Replication: SLE	563	192,504	1.09	0.41	-	-	-	-	-	-	-	-
	Replication: Multi-trait (PsO-SLE)	1,139	192,761	0.99	0.86	-	-	-	-	-	-	-	-
rs2210366 (*HBS1L*)	GWAS: Multi-trait (AD-BA-GD-PO-RA-T1D)	19,609	142,192	1.07	$2.5{\times}10^{-8}$	86,112	291,471	1.01	0.03	105,721	433,663	1.03	$2.9{\times}10^{-6}$
	Replication: PsO	576	192,578	1.01	0.83	11,231	503,713	0.98	0.33	11,807	696,291	0.99	0.37
	Replication: SLE	4,785	200,935	1.00	0.94	5,202	511,575	1.03	0.20	9,987	712,510	1.02	0.34
	Replication: Multi-trait (PsO-SLE)	5,361	201,192	1.00	0.88	16,417	511,575	1.00	0.86	21,778	712,767	1.00	0.93
rs4529910 (*POU2AF1*)	GWAS: Multi-trait (AD-BA-GD-PO-RA-T1D)	19,609	142,192	0.96	$8.3{\times}10^{-4}$	86,112	291,471	0.96	$5.7{\times}10^{-8}$	105,721	433,663	0.96	$1.9{\times}10^{-10}$
	Replication: PsO	576	192,578	0.92	0.19	11,231	503,713	0.95	$\mathbf{6.5{\times}10^{-4}}$	11,807	696,291	0.95	$\mathbf{2.9{\times}10^{-4}}$
	Replication: SLE	4,785	200,935	0.99	0.61	5,202	511,575	0.96	0.12	9,987	712,510	0.97	0.14
	Replication: Multi-trait (PsO-SLE)	5,361	201,192	0.98	0.32	16,417	511,575	0.95	$\mathbf{2.1{\times}10^{-4}}$	21,778	712,767	0.96	$\mathbf{1.9{\times}10^{-4}}$

† *P*-value in the fixed effect model.

AD, atopic dermatitis; BA, bronchial asthma; GD, Grave’s diseases; PO, pollinosis; RA, rheumatoid arthritis; T1D, type 1 diabetes; PsO, psoriasis; SLE, systemic lupus erythematosus

P-values satisfying the threshold of 0.05 and consistent effect with the original GWAS multi-trait analysis (AD-BA-GD-PO-RA-T1D) are shown in bold.

Shirai Y, *et al. Ann Rheum Dis* 2022; 81:1301–1312. doi: 10.1136/annrheumdis-2022-222460
